# Aerobic bacteria produce nitric oxide via denitrification and promote algal population collapse

**DOI:** 10.1038/s41396-023-01427-8

**Published:** 2023-05-12

**Authors:** Adi Abada, Roni Beiralas, Delia Narvaez, Martin Sperfeld, Yemima Duchin-Rapp, Valeria Lipsman, Lilach Yuda, Bar Cohen, Raanan Carmieli, Shifra Ben-Dor, Jorge Rocha, Irene Huang Zhang, Andrew R. Babbin, Einat Segev

**Affiliations:** 1 Department of Plant and Environmental Sciences, The Weizmann Institute of Science, Rehovot, Israel; 2 Depertment of Chemical Research Support, The Weizmann Institute of Science, Rehovot, Israel; 3 Department of Life Science Core Facilities, The Weizmann Institute of Science, Rehovot, Israel; 4 CIDEA Consortium Conacyt-Centro de Investigación en Alimentación y Desarrollo, Hermosillo, Mexico; 5 Department of Earth, Atmospheric and Planetary Sciences, Massachusetts Institute of Technology, Cambridge, MA USA

**Keywords:** Water microbiology, Biogeochemistry

## Abstract

Microbial interactions govern marine biogeochemistry. These interactions are generally considered to rely on exchange of organic molecules. Here we report on a novel inorganic route of microbial communication, showing that algal-bacterial interactions between *Phaeobacter inhibens* bacteria and *Gephyrocapsa huxleyi* algae are mediated through inorganic nitrogen exchange. Under oxygen-rich conditions, aerobic bacteria reduce algal-secreted nitrite to nitric oxide (NO) through denitrification, a well-studied anaerobic respiratory mechanism. The bacterial NO is involved in triggering a cascade in algae akin to programmed cell death. During death, algae further generate NO, thereby propagating the signal in the algal population. Eventually, the algal population collapses, similar to the sudden demise of oceanic algal blooms. Our study suggests that the exchange of inorganic nitrogen species in oxygenated environments is a potentially significant route of microbial communication within and across kingdoms.

## Introduction

**Fig. 1:**
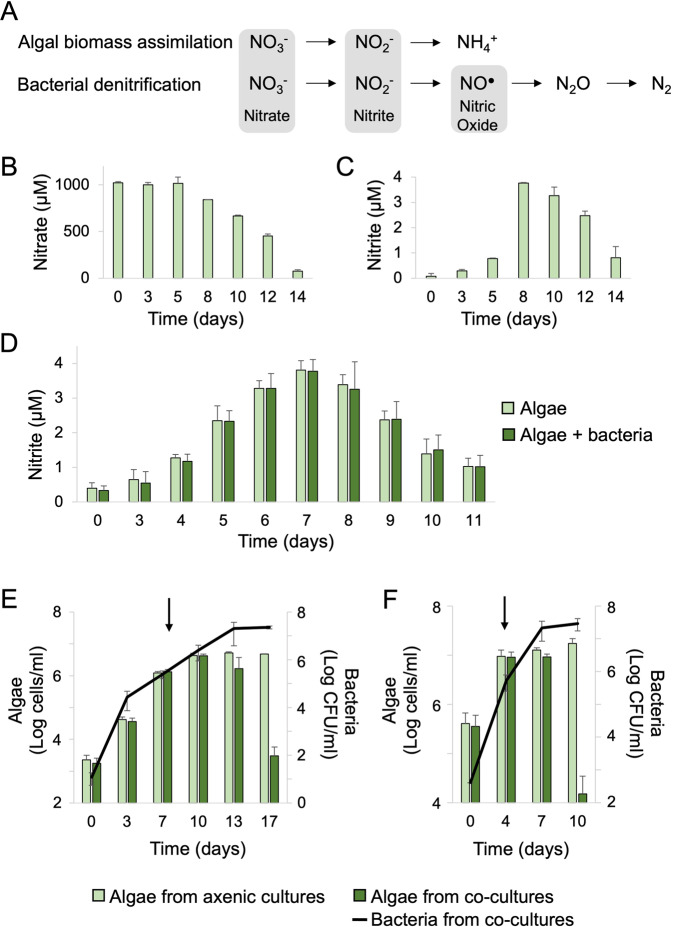
Algal nitrite secretion during exponential growth is linked to the timing of algal death in co-cultures. **A** Assimilatory nitrate reduction to ammonium during algal biomass assimilation produces nitrite as an intermediate. Dissimilatory nitrate reduction to di-nitrogen during bacterial denitrification also includes nitrite as an intermediate, which is further reduced to NO. The main nitrogen species discussed in the manuscript are shaded in gray. **B** Nitrate and (**C**) nitrite detected in filtrates of axenic algal cultures on the indicated days. **D** Daily monitoring of nitrite in filtrates of axenic algal cultures (light green) and co-cultures (dark green). **E**, **F** Algal growth (bars) and bacterial growth (black line) in axenic algal cultures (light green bars) and algal-bacterial co-cultures (dark green bars). **E** Cultures initiated with an algal inoculum of 10^3^ algae/ml. **F** Cultures initiated with an algal inoculum of 10^5^ cells/ml. Black arrows indicate the day of the extracellular nitrite peak as shown in (D) and in Fig. S1D. Each data point in the figure consists of at least 3 biological replicates, error bars designate ±SD. Statistical significance was calculated using a two-sample *t* test to compare algal cell counts in axenic cultures versus in co-cultures. **E** Algal cell counts on day 17 and (F) on day 10 were significantly different between axenic and co-cultures and resulted in *p* < 0.01.

Various marine bacteria that inhabit oxygenated surface waters carry denitrification genes. This is surprising, as denitrification is a microbial process which allows microorganisms to maintain cellular bioenergetics in limiting oxygen concentrations [1]. To conduct denitrification, specialized microbes express enzymes that are responsible for a multi-step reduction process of nitrogen species that serve as terminal electron acceptors. The full denitrification process commences with the reduction of nitrate (NO_3_¯) to nitrite (NO_2_¯), then to nitric oxide (NO), to nitrous oxide (N_2_O) and finally to dinitrogen (N_2_) (Fig. 1A). In the ocean, microbial denitrification widely occurs in oxygen deficient zones (ODZs) and ocean sediments [2, 3], and all denitrification intermediates including nitrite, NO and nitrous oxide, are observed to accumulate in the marine environment [3–5]. Knowledge regarding the NO intermediate is scarce due to its short-lived nature [3]. Therefore, its cryptic presence in ocean waters might have overlooked consequences.

NO in the ocean may originate from sources other than denitrification. In fact, most living organisms produce this molecule for signaling, both under normal and perturbed physiology [6–8]. NO is often associated with processes of programmed cell death (PCD) and oxidative stress, both in unicellular and multicellular organisms [9–12]. Since NO is a small, membrane-permeable gas molecule, it passes easily through adjacent cells [13]. The short half-life of NO in oxygenated environments ensures a localized response within tissues [13]. Similarly, NO in unicellular organisms can stimulate a response in a close neighbor [14–16]. Indeed, NO has been suggested to diffuse between cells in a dense population of the microalga *Gephyrocapsa huxleyi* (formerly *Emiliania huxleyi* [17]) [18].

*G. huxleyi* is the most widespread coccolithophore in modern oceans [19]. Coccolithophores are unicellular marine algae that cover their cell with intricate discs made of crystalline calcium carbonate. *G. huxleyi* forms vast annual blooms that stretch over thousands of square kilometers of ocean surface [20, 21]. The blooms gradually form over several weeks and then suddenly collapse [22–24]. NO has been shown to be associated with bloom demise, particularly as a result of viral infection [18]. Notably, algal populations that exhibit NO production in the ocean and in the lab also harbor a rich bacterial community. Among these bacteria are the Roseobacters, an abundant and metabolically versatile group of marine bacteria that are commonly found associated with microalgae [25–29]. The *Rhodobacteraceae* family contains both marine and non-marine bacteria, the term ‘Roseobacter group’ is an operational definition referring only to marine members of the family [30]. Many Roseobacter bacteria carry denitrification genes [31], and thus have the potential to generate NO and contribute to the observed NO-related algal demise. However, many members of this bacterial group are known aerobes (Table S1), thus the presence of denitrification genes in their genomes is puzzling.

Here, we used an algal-bacterial model system comprised of the alga *G. huxleyi* and the Roseobacter bacterium *Phaeobacter inhibens* [29] to study microbial inorganic nitrogen exchange. The interaction between *G. huxleyi* and *P. inhibens* is dynamic; initially algae and bacteria exchange beneficial molecules in a mutualistic phase [29]. However, as the culture ages, bacteria become pathogenic and kill their algal partners [29]. Here we show that the aerobic Roseobacter *P. inhibens* activates denitrification genes and produces NO under well-oxygenated conditions. Our data revealed that *P. inhibens* bacteria generate NO by reducing nitrite, and that nitrite is secreted by the alga *G. huxleyi* during algal exponential growth. Furthermore, we demonstrate that bacterial NO is detected extracellularly, and is involved in promoting a PCD-like process in algal populations. During their demise, algae further generate and release NO, thereby propagating the signal among the algal population. Importantly, analysis of environmental metagenomes, metatranscriptomes and metagenome-assembled genomes (MAGs) from oxygen-rich waters support that bacterial denitrification genes and transcripts co-occur in association with phytoplankton, highlighting the potential ecological relevance of our laboratory findings. Our results unveil an algal-bacterial chemical exchange mediated through inorganic nitrogen species and point to NO as an inter-kingdom signaling molecule. These observations have implications for many interactions between eukaryotic hosts and bacteria that harbor denitrification genes for purposes other than anaerobic energy production.

## Results

### Algae secrete nitrite in a growth phase-dependent manner

Algal exudates of *G. huxleyi* support the growth of the bacterium *P. inhibens* in seawater [29]. While it is commonly known that algal nutrients can support the growth of heterotrophic bacteria [32], the identity and quantity of inorganic nutrients that are secreted by specific algae remain unknown. To better understand the algal-bacterial interaction, we measured the main inorganic nutrients that are secreted by growing algal cultures. Our data indicate that the levels of nitrate (NO_3_¯) and phosphate (PO_4_^3^¯) in the medium gradually decreased over time (Figs. 1B, S1A), indicative of assimilation by the growing algal population. As algae are primary producers, they can uptake inorganic nitrogen from seawater in the form of nitrate, reduce it to nitrite (NO_2_¯) and further reduce it in an assimilatory pathway to generate ammonium (NH_4_^+^) (Fig. 1A). Surprisingly, we observed an extracellular peak of the intermediate nitrite (Fig. 1C). Nitrite was previously reported to leak from diatoms, another microalgal group, during exponential growth. The exuded nitrite can later be up-taken by cells at stationary phase [33–35]. Contrary to the nitrite peak, ammonium and sulfate (SO_4_^2^¯) levels remained unchanged in the medium (Fig. S1B, C).

To gain improved temporal resolution of algal nitrite secretion, we monitored daily the extracellular levels of nitrite in axenic algal cultures. The release of nitrite was detected during algal exponential growth, and the highest nitrite concentration, which represents the buildup of nitrite released during algal growth, was detected at the end of algal exponential growth phase (Fig. 1D, E). To further characterize the association between algal growth and nitrite dynamics, we initiated algal cultures with a high algal inoculum (10^5^ cells/ml) (Fig. 1F, Fig. S1D). A denser algal inoculum results in expedited exponential growth and an earlier stationary phase (Fig. 1F). Consequently, in cultures initiated with a denser inoculum, the nitrite peak was detected earlier than in cultures that were initiated with a more dilute inoculum (Fig. S1D). Taken together, these observations suggest that nitrite secretion is indicative of an exponentially growing algal population.

Bacteria substantially impact algal physiology and influence growth dynamics of algal populations [29, 36–38]. We therefore explored whether co-culturing algae with bacteria would influence nitrite secretion. The concentration and timing of the nitrite peak was notably similar in axenic algal cultures and algal-bacterial co-cultures, regardless of the initial algal inoculum (Fig. 1D, Fig. S1D). These findings suggest that nitrite secretion by algae is not influenced by the presence of bacteria and remains an indicator of algal growth phase in co-cultures.

### Algal death in algal-bacterial co-cultures occurs following the algal nitrite peak

Bacteria become pathogenic towards aging algae in co-cultures (Fig. 1E) [29], but it is yet unknown how bacteria sense the aging of their algal host. Therefore, we examined whether algal-secreted nitrite can serve as a signal for bacteria to become pathogens (manifested by algal death). In line with this idea, changing the timing of the nitrite peak is expected to result in a concomitant shift in the timing of sudden algal death. Timing of nitrite secretion can be altered by adjusting the density of the algal inoculum (Fig. S1D), therefore we initiated algal-bacterial co-cultures with a denser algal inoculum. As can be seen in Fig. 1F, a denser algal inoculum resulted in earlier algal death, while the concentration of nitrite at the peak of secretion remained similar compared to cultures that were initiated with a more dilute algal inoculum. These results propose a correlation between the growth phase of the algal population, indicated by the peak of algal nitrite, and the subsequent bacterially triggered algal death. Taken together, algal nitrite might signal bacteria to become pathogenic.

### Bacteria harbor denitrification genes related to nitrite metabolism

To explore whether secreted algal nitrite serves as a signaling molecule for bacteria, we investigated the genetic potential of *P. inhibens* to detect and metabolize nitrite. A search for bacterial functions related to nitrite metabolism identified five genes in the *P. inhibens* genome (Fig. S2A). Three are nitrite reductases; *nirK*, which has been extensively studied in the context of nitrite reduction to NO in denitrifying bacteria [39], and two putative nitrite reductase/sulfite oxidase enzymes (*msrPQ* and Molybdopterin-dependent oxidoreductase), with a previous study indicating that they indeed reduce nitrite [40]. The two other detected genes are a nitrite transporter, related to nitrite uptake, and a protein containing a nitrate/nitrite sensing domain with no suggested activity. We further examined the genomic locus of *nirK* in the published genome of *P. inhibens* DSM17395 and found multiple genes and operons that were previously annotated as related to denitrification: (i) an adjacent hypothetical protein similar to a *nirV* gene. The *nirV* gene is commonly found in the *nirK* operon but its activity is yet unknown [41], (ii) a *nor* operon encoding an NO reductase (*norCBQD*), (iii) a *nnrS* gene that is suggested to help alleviate NO-related stress in bacteria [42] and (iv) a *nnrR* gene encoding an established NO transcriptional regulator [43, 44] (Fig. S2B, Table S2). These denitrification-related functions are all found on a single bacterial 262 kb plasmid, one of three native plasmids of *P. inhibens* [45].

Denitrification is a common metabolic process in anaerobic bacteria that reduce inorganic nitrogen species as terminal electron acceptors for respiration (Fig. 1A) [1]. *P. inhibens* specializes in interactions with photosynthesizing hosts that produce oxygen [29], therefore the function of the denitrification genes in this bacterium is curious. We examined the ability of *P. inhibens* bacteria to grow under oxygen-depleted conditions by utilizing nitrite as terminal electron acceptor (Fig. 1A). Our results demonstrated that *P. inhibens* exhibited very poor growth under anoxic conditions (Fig. S2C), while the known denitrifier strain *Phaeobacter inhibens* T5 (DSM16374) grew well under these conditions [46]. The poor growth of *P. inhibens* DSM 17395 under our experimental conditions could be due to residual oxygen levels or could represent actual poor growth using the denitrification pathway. The poor growth in an anoxic environment suggests that denitrification genes in this bacterium may possess additional roles other than bioenergetics.

### Bacteria can produce and secrete nitric oxide under oxic conditions

**Fig. 2:**
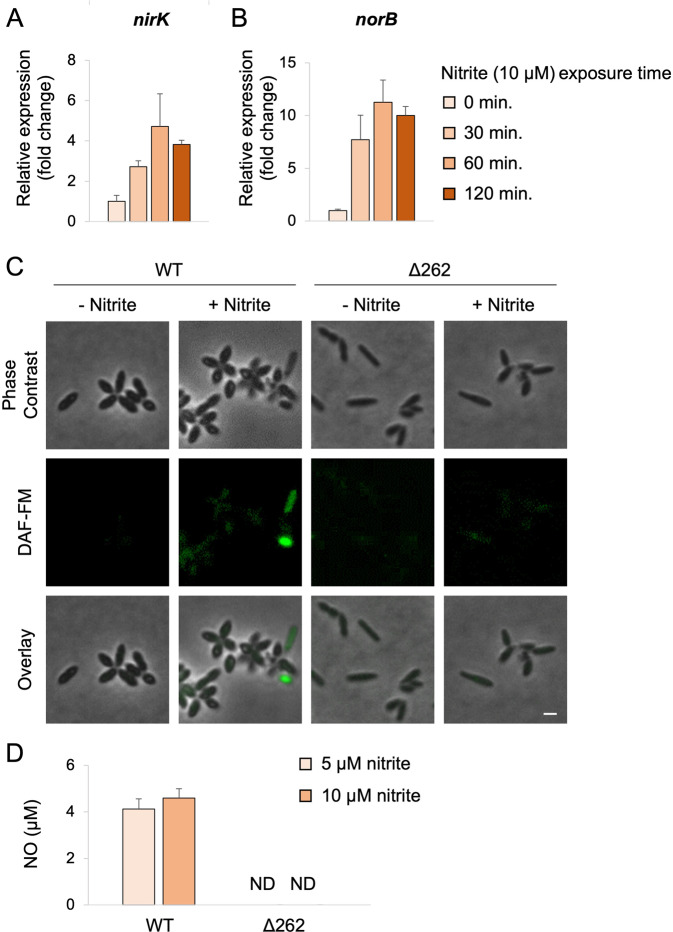
Bacteria produce and secrete NO under oxic conditions. Relative gene expression of *P. inhibens* denitrification genes under exposure to 10 µM nitrite for the indicated times; (**A**) *nirK*, (**B**) *norB*. Each data point consists of 3 biological replicates, error bars designate ±SD. **C** Microscopy images of WT and Δ262 bacteria cured from the native 262 kb plasmid, stained with the fluorescent NO-indicator diacetate DAF-FM, and incubated with or without 100 µM nitrite for 2 h. Scale bar corresponds to 1 µm. **D** Extracellular nitric oxide concentrations of WT and Δ262 bacteria incubated with the indicated nitrite concentrations. Each data point consists of 3 biological replicates, each containing 10^7^ cells/ml. Error bars designate ±SD. ND stands for not detected.

*G. huxleyi* cells secrete nitrite during exponential growth (Fig. 1D) and P*. inhibens* bacteria have the genetic potential to reduce nitrite to NO and further to N_2_O through denitrification (Fig. S2A, B). NO was previously shown to play key roles in algal physiology during viral infection [18, 47] and in response to specific aldehydes [48, 49], and it is known to be central in PCD in multicellular organisms [6–8]. We therefore explored the role of NO in the *G. huxleyi - P. inhibens* interaction. First, we examined whether denitrification genes are expressed in pure bacterial cultures when exposed to exogenous nitrite under oxic conditions, mimicking the nitrite secreted by algae. Our data show that immediately following the addition of nitrite the expression of bacterial *nirK* and *norB* increased, reaching an expression peak of fourfold and tenfold, respectively, within one hour of exposure (Fig. 2A, B). The enzyme encoded by *nirK* produces NO by reducing nitrite while the enzyme encoded by *norB* further reduces NO to N_2_O (Fig. 1A). Next, we examined whether the enzyme encoded by *norB* is involved in alleviating stress that might be induced by NO production. Therefore, we deleted the *nor* operon (Table S2, rows 4–7) in *P. inhibens*, and tested if the mutant was more sensitive to the NO donor DEANO (Diethylammonium (Z)-1-(N,N-diethylamino)diazen-1-ium-1,2-diolate) or to nitrite compared with wild-type (WT) bacteria (Fig. S2D). We found no detectable difference in the growth of WT and *Δnor* bacteria when exposed to exogenous nitrite or NO. This result suggests that the absence of NorB does not result in detectable stress because of exposure to denitrification intermediates.

Since the denitrification genes of *P. inhibens* are expressed following the addition of nitrite, we wanted to test production of NO in bacterial cells exposed to nitrite under oxic conditions. To this end, intracellular NO production in bacterial cells was tracked using DAF-FM Diacetate (4-Amino-5-Methylamino-2’,7’-Difluorofluorescein Diacetate), a fluorescent probe that emits a signal upon intracellular NO binding. Our results demonstrate that the addition of nitrite to pure bacterial cultures results in increased levels of intracellular NO (Fig. 2C). To validate that increased NO production is dependent on the expression of denitrification genes, we examined a bacterial mutant that was cured of its native 262 kb plasmid (Δ262), and therefore no longer carries any denitrification genes [45]. Our results indicate that under the same experimental conditions, the bacterial mutant Δ262 grows to the same optical density as the WT (Fig. S2E) but does not produce NO (Fig. 2C). According to the microscopy images, the intracellular production of NO in WT bacteria was heterogeneous (Fig. 2C). Heterogeneity within the bacterial population appears to be a broad phenomenon associated with many diverse functions [50]. At present, it remains to be uncovered what is the regulatory process that gives rise to this heterogeneity, and what are the ecological implications of bacterial heterogeneous NO production.

If bacterial NO diffuses out of the producing bacterial cell to the extracellular environment, it can potentially reach and affect neighboring algal cells, especially given the close algal-bacterial proximity previously reported [29]. To explore this idea, we first assessed whether bacterial NO can be detected extracellularly. Therefore, we utilized the Liposome-Encapsulated-Spin-Trap (LEST) method that was previously developed to measure extracellular NO secreted by microorganisms to their surroundings [47], and was successfully applied in microalgal cultures [18, 47]. This method utilizes liposomes that continuously absorb and stabilize the NO that is secreted during several hours. Therefore, the short NO half-life does not influence the measurement. Extracellular NO was indeed detected in the medium of bacterial cells supplemented with nitrite, but not in the medium of mutant Δ262 bacteria (Fig. 2D). Taken together, our data demonstrate that *P. inhibens* bacteria produce and secrete NO upon exposure to extracellular nitrite under oxic conditions. The fact that the NO intermediate is detected extracellularly, even though the *norB* gene is expressed, indicates that at least some NO escapes intracellular catalytic reduction.

### NO production by bacteria is involved in triggering algal death

**Fig. 3:**
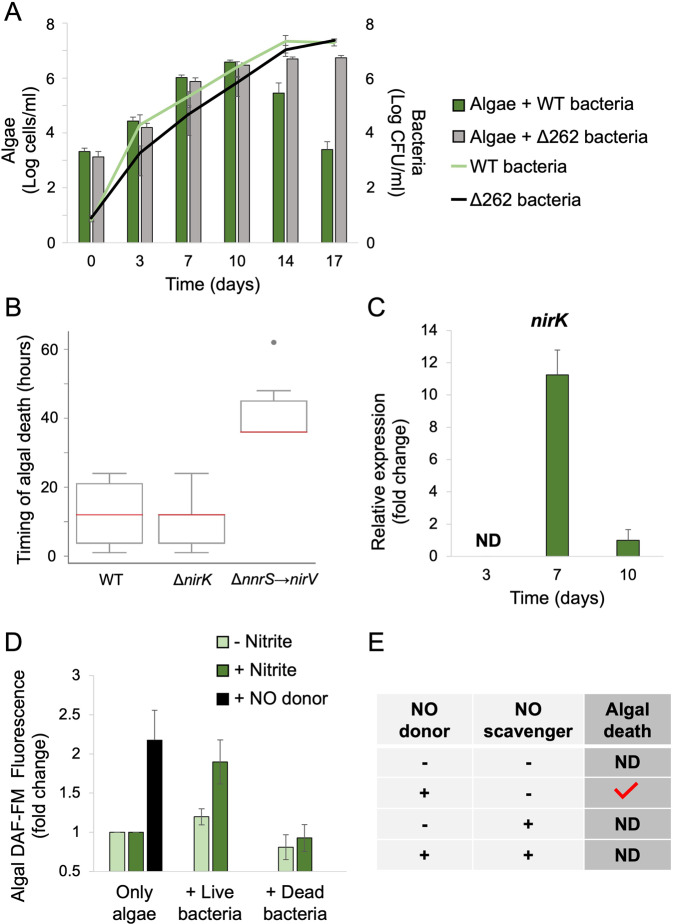
Bacterial NO production by denitrification is involved in triggering algal death. **A** Algal growth (bars) and bacterial growth (lines) in co-cultures with WT bacteria (green bar and line) and Δ262 bacteria cured from the native 262 kb plasmid (gray bar and black line). Each data point consists of 3 biological replicates, error bars designate ±SD. Statistical significance was calculated using a two-sample *t* test to compare algal cell counts in co-cultures with WT bacteria versus in co-cultures with Δ262 bacteria. Algal cell counts were significantly different on day 14 and 17 and resulted in *p* < 0.01. Statistical significance was calculated using a two-sample *t* test to compare the growth of WT bacteria versus Δ262 bacteria in co-cultures and did not result in significant differences. **B** Algae were co-cultured with either WT, Δ*nirK* or Δ*nnrS→nirV* bacteria. Co-culturing was conducted as described in materials and methods. Algal death was monitored manually every 6–8 h and death was defined as the complete change of the culture color from green to white. The timing of algal death was determined as follows: Co-cultures grew for 10 days and then algal death was monitored. The first culture that exhibited algal death was enumerated as 1 h, and the timing of algal death in all other co-cultures was measured from that point on (i.e. a culture that exhibited algal death 48 h after the first culture, was enumerated as 48). Each type of co-culture (with WT, Δ*nirK* or Δ*nnrS→nirV* bacteria) included 6 biological replicates. Box-plot elements are: center line—median; box limits—upper and lower quartiles; whiskers—min and max values, point—outlier. Statistical significance was calculated using a two-sample *t* test to compare timing of algal death between co-cultures with WT versus Δ*nirK* bacteria and between WT versus Δ*nnrS→nirV* bacteria. The difference between the timing of algal death in co-cultures with WT versus Δ*nnrS→nirV* bacteria was significant and resulted in *p* < 0.01. **C** Relative expression of *nirK* in bacterial cells from an algal-bacterial co-culture (corresponding to co-cultures in **A**), sampled on the indicated days. Data is relative to the expression on day 10. Each data point consists of 4 biological replicates, error bars designate ±SD. ND stands for not detected. **D** Fluorescence of algal cells stained with the fluorescent NO-indicator diacetate DAF-FM, incubated with live or dead bacteria for 2 h under the following conditions: control (– nitrite, light green), exposure to nitrite ( + nitrite [10 µM], dark green), or exposure to a chemical NO donor ( + NO donor, [300 µM] DEANO, black). Results represent 3 biological replicates, each containing 10,000 algal cells and 10^7^ bacterial cells when indicated. Error bars designate ±SD. Statistical significance was calculated using a two-sample *t* test to compare DAF-FM fluorescence between treatments. DAF-FM fluorescence between algal treatment with or without nitrite versus NO donor treatment was significant and resulted in *p* < 0.01. DAF-FM fluorescence of algae treated with live bacteria with nitrite versus without nitrite was significant and resulted in *p* < 0.01. **E** The effect on the death of axenic algal cultures upon adding (+) a NO donor (a single dose of 100 µM DEANO) and a NO scavenger (20 µM c-PTIO). Red checkmark indicates observed algal death. Results represent at least 3 biological replicates. ND stands for not detected.

The temporal and metabolic link between algal nitrite secretion and bacterial NO production, together with the subsequent algal death in co-cultures, point to the possible involvement of bacterial NO in algal death. If bacterial denitrification intermediates are involved in promoting algal death, the mutant strain Δ262 should not be able to trigger algal death in co-cultures. Indeed, algae that were co-cultivated with Δ262 bacteria did not exhibit algal death (Fig. 3A). Importantly, WT and Δ262 bacteria exhibit similar growth curves both in pure bacterial cultures and co-cultures (Fig. 3A, Fig S2E). Of note, the 262 kb plasmid harbors genes other than the denitrification genes [51, 52]. To specifically reveal the function of bacterial denitrification genes in promoting algal death, we deleted the denitrification locus from the plasmid- a region of 10.4 kb containing all denitrification genes (Fig. S2B, Table S2). Co-cultivation of algae with these denitrification-deficient bacteria resulted in a marked delay in algal death (Fig. 3B), although algal death was not prevented. A targeted deletion of only the *nirK* gene did not result in a detectable impact on algal death (Fig. 3B). In both mutants, we did not detect intracellular NO using the DAF-FM dye (Fig. S3). It is currently unknown whether additional nitrite reductases outside of the denitrification locus (such as two putative nitrite reductase/sulfite oxidase enzymes discussed earlier) are regulated in trans by denitrification regulators. This would explain why a deletion of the *nirK* reductase did not influence bacterial pathogenicity while deletion of the entire locus exhibited a marked impact. In line with this idea, the NnrR regulator, which is encoded by the denitrification locus, was previously shown to regulate in trans over 170 genes, revealing previously unrecognized components of the denitrification pathway [53]. Our results demonstrate that the entire bacterial denitrification locus is involved in promoting algal death, though additional components outside of this locus are likely to exist. Moreover, it appears that redundant routes of bacterial pathogenicity are executed in parallel. Previous reports from our group demonstrated another route of pathogenicity involving a phytohormone that is secreted by bacteria and promotes algal death [29]. The full repertoire of pathogenic pathways in *P. inhibens* is not yet known. Revealing all components involved in aerobic bacterial denitrification and understanding how they act in concert with additional pathogenicity routes, is currently studied in our lab.

While other nitrite reductases might be involved, the Nirk is the most characterized reductase, and thus we further examined the expression of *nirK* in algal-bacterial co-cultures. Expression of this bacterial gene in co-cultures would suggest bacterial NO production through reduction of algal-secreted nitrite. Our results show more than 10-fold increase in bacterial *nirK* expression in co-cultures during the algal exponential growth phase (Fig. 3C), compared to expression during algal stationary phase. The increased *nirK* expression is concurrent with the algal nitrite peak (Fig. 1D, E). We were not able to detect *nirK* expression in co-cultures during earlier culturing stages, either because it is not expressed or due to low bacterial numbers. These results demonstrate the co-occurrence of the algal-secreted nitrite peak, and the response of bacteria that express genes to reduce nitrite to NO in co-cultures.

Bacteria secrete NO when exposed to nitrite (Fig. 2D), and NO is known to diffuse across membranes from producing cells to neighboring cells [13]. We therefore tested the ability of bacterial NO to diffuse from NO-producing bacteria to adjacent algal cells. We monitored intracellular NO in algal cells that were exposed to an extracellular chemical NO donor, or to bacteria. Our results show that when algae were stained with the fluorescent NO indicator DAF-FM diacetate, exposure to an extracellular chemical NO donor resulted in increased fluorescence (Fig. 3D). This observation indicates that external NO can indeed diffuse into algal cells where it binds the fluorescent indicator. Moreover, when algae were incubated with bacteria, and supplemented with nitrite, increased fluorescence was also evident (Fig. 3D). A similar experiment conducted with dead bacteria or without nitrite addition, did not yield increased fluorescence (Fig. 3D). Thus, it appears that live bacteria reduce nitrite and generate NO that can diffuse into adjacent algal cells.

We next examined whether the actual NO molecule triggers algal death. If extracellular NO, secreted by bacteria, causes algal death in the algal-bacterial interaction, then treating axenic algal cells with extracellular chemical NO should result in similar sudden algal death. Indeed, addition of a chemical NO donor to axenic algal cultures resulted in death of the cultures. Algal death was observed both when a single dose of NO was applied (100 μM at late exponential or at stationary phase seen in Fig. 3E and 10, 50 or 100 μM at early exponential phase seen in Fig. S4A) and under semi-continuous application (1.5–15 μM twice a day seen in Fig. S4D). This effect was rescued by the addition of the chemical NO scavenger c-PTIO (Fig. 3E), indicating that extracellular NO can trigger algal death.

Our data indicate that the algal response to external NO depends on the algal growth phase, the NO concentration, and the duration of exposure (Fig. S4). Nanomolar concentrations of NO were previously shown to promote the growth of *G. huxleyi* [54]. A slight stimulatory impact is also seen in our data under 1 μM treatment (Fig. S4 panels A and C). This stimulatory influence of NO might explain increased algal growth that was previously observed in co-cultures prior to algal death [29]. Thus, the transition between the stimulatory to inhibitory impact on algal growth in co-cultures could be the result of low versus high levels of NO produced by bacteria in response to corresponding levels of nitrite exuded by algae.

### Extracellular NO triggers a PCD-like death in algal cells

**Fig. 4:**
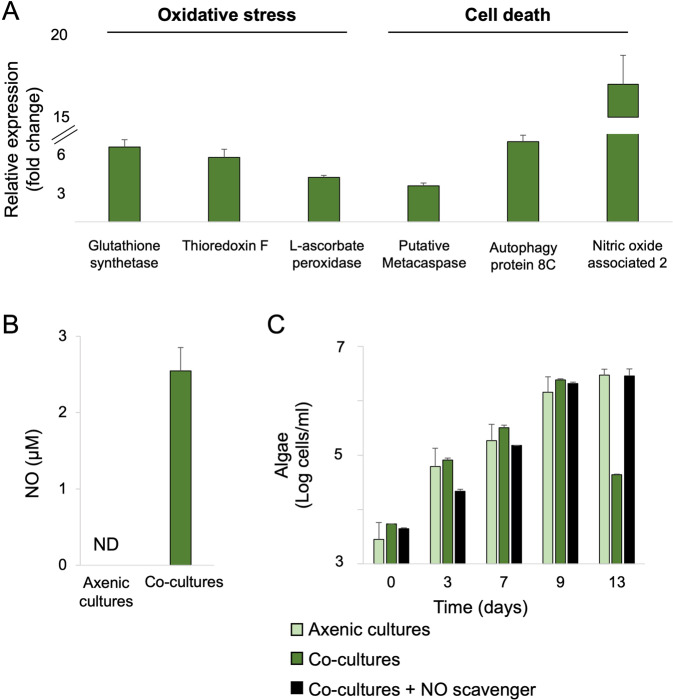
Extracellular NO triggers a PCD-like process in algae, followed by algal NO production and secretion. **A** Relative gene expression of algal oxidative stress and PCD-like genes following incubation with a NO donor ([100 µM] DEANO for 18 h). Annotated gene products are depicted (Table 4). **B** Extracellular NO concentrations of axenic algal cultures and algal-bacterial co-cultures on day 10. **C** Algal growth in axenic algal cultures (light green), co-cultures (dark green) and co-cultures supplemented with a NO scavenger ([20 µM] c-PTIO added on day 9 of the co-culture, black). All data points in the figure consist of 3 biological replicates, error bars designate ±SD. Statistical significance was calculated using a two-sample *t* test to compare algal cell counts on day 13 in co-cultures versus algal cell counts in co-cultures treated with NO scavenger. Algal cell counts were significantly different and resulted in *p* < 0.05.

Recent evidence suggests that environmental stresses, viral infection, and bacterial cues can trigger oxidative stress and a process similar to programmed cell death (PCD) in *G. huxleyi* [11, 29, 55]. It was demonstrated that algae undergo a PCD-like process when algal death is triggered by bacteria [29, 56]. Several PCD-like and oxidative stress-related genes are upregulated in algae during bacterial-triggered algal death [29]. We reasoned that if bacterial NO is the inducer of algal death in co-cultures, then treatment with extracellular NO should promote the expression of similar genes in algal cultures. To explore this possibility, we first compiled an updated list of algal genes that are upregulated during bacterial-triggered algal death. Recently, a novel *G. huxleyi* transcriptome was generated by our lab, specifically designed to reveal expression of genes involved in algal-bacterial interactions [57]. Mining the novel transcriptome revealed various algal genes that are upregulated during algal death in co-culture and therefore are putatively involved in PCD and oxidative stress (Fig. S5A). It has been previously shown in microalgae that there are multiple routes of PCD which trigger different PCD genes [58]. Therefore, to compile our “PCD-fingerprint” genes we chose two additional genes from a previous study [29], thereby using two independent datasets to define genes indicative of PCD. Although some of the selected genes are general hallmarks of algal cell death [11, 55], the combination of genes was used as an indicator of a PCD-like algal process that is induced by bacteria. Our data show that treatment of axenic algal cultures with a chemical NO donor, mimicking bacterial NO secretion in co-culture, results in increased expression of 3–8-fold of the selected genes that serve as proxy for bacterially triggered algal death (Fig. 4A).

### Algae produce nitric oxide that propagates through the algal population

Algae were previously shown to produce and secrete NO [18, 48]. Since NO appears to be central in the algal-bacterial interaction, we characterized NO production and propagation in the algal population. In plants and microalgae, collectively termed photoautotrophs, NO production, its roles, and regulation are not well understood. In several photoautotrophs, NO generation appears to involve a GTP-binding Nitric Oxide-Associated protein (NOA) [49, 59]. Nitric oxide synthase (NOS) activity has been demonstrated in several photoautotrophs [48, 60] but the NOS encoding gene was only found in the green alga *Ostreococcus tauri* [61]. Enzymatic reduction of nitrite to NO by nitrate reductase (NR) was also shown to produce NO in photoautotrophs [47, 62, 63].

We found two *noa* genes in *G. huxleyi*; *noa1*- similar to the gene sequence of diatom *noa* (MZ773649) [49] and *noa2*- similar to the plant *noa* gene sequence (MZ773650) [59] (Fig. S5B, Table S3). We did not detect *nos* genes, and our results suggest that NR-mediated production of NO is unlikely, as addition of nitrite to algae did not result in NO production (Fig. 3D). While the role of NOA in NO metabolism is not yet resolved, our data indicate that when axenic algal cultures were treated with a chemical NO donor, the expression of the *noa2* gene increased over 15-fold (Fig. 4A). In addition, transcriptomic data from co-cultures indicate that the expression of the *noa2* gene increased during stationary phase and algal death, while the expression of *noa1* did not change (Fig. S5C) [57]. Further genetic and biochemical characterization will be needed to reveal the role of the *G. huxleyi noa* genes and to understand the algal NO-producing machinery.

Algal *noa* genes are expressed during co-culture, therefore we tested whether algae produce NO in algal-bacterial co-cultures. We detected extracellular NO only during the stationary phase of co-cultures, but not in axenic algal cultures of the same age (Fig. 4B). To investigate whether NO is produced by algae upon exposure to external NO, the production and propagation of NO in the algal population was visualized. We treated algae with a chemical NO donor, then thoroughly washed the chemical treatment, and seeded an inoculum of the treated algae into a fresh algal culture stained with the fluorescent NO indicator DAF-FM. Our results show that NO is produced by increasing numbers of algal cells following seeding (Movie S1). Similar propagation of NO was previously observed in a diatom population exposed to a specific aldehyde [48]. Our observations demonstrate that algae produce NO upon exposure to external NO, thus propagating the signal in the algal population.

It is possible that our previous observation of increased intracellular NO in algal cells exposed to NO (Fig. 3D), is due to algal NO production in response to the external NO (in addition to diffusion of the treatment into the cell). Taken together, our data imply that algal death can be triggered by bacterial NO, and this exogenous NO promotes further NO production and secretion by algae. Therefore, it stands to reason that a NO scavenger should rescue algal death in algal-bacterial co-cultures. Indeed, addition of a NO scavenger to co-cultures immediately before the population reaches stationary phase (when the peak of extracellular NO was measured, Fig. 4B) completely prevented algal death (Fig. 4C). Taken together, NO secretion by algae can propagate the death signal among algal cells and promote collapse of the algal population.

### Denitrification genes and transcripts are detected in oxygenated regions of the ocean

**Fig. 5:**
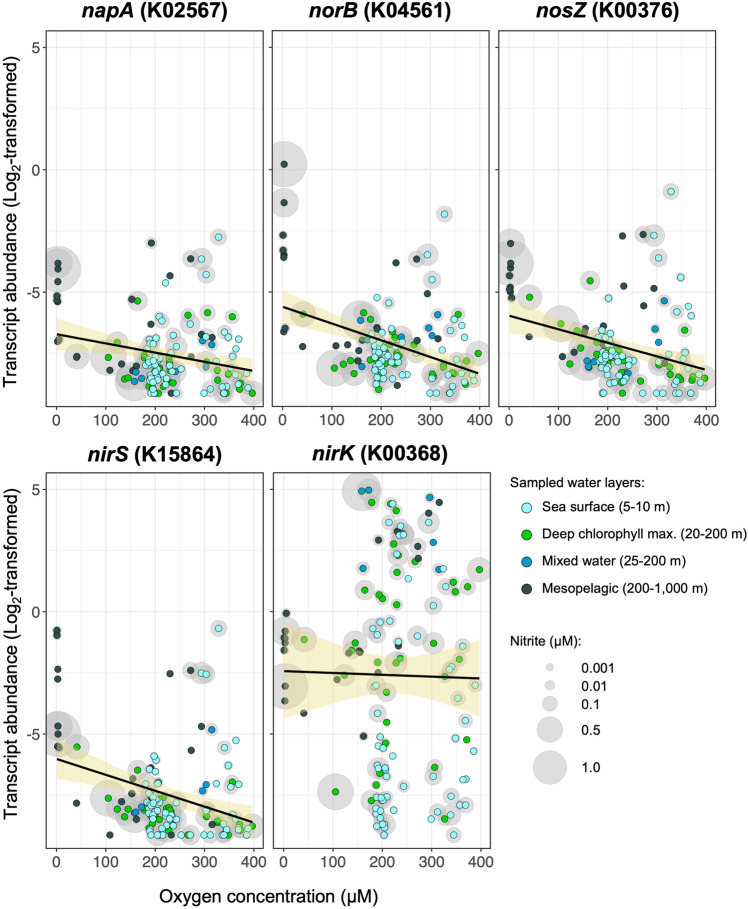
Expression of *nirK* in oxygenated marine environments. Transcript abundances of selected denitrification genes (*napA, norB, nosZ* and *nirS*) decreased with increasing oxygen concentrations. In contrary, transcript abundances of *nirK* were frequently high in phytoplankton-rich water layers (represented by the deep chlorophyll maximum, green dots) in which oxygen levels of 100–400 µM were detected, likely produced by phytoplankton. In the same samples, nitrite levels of up to 1.5 µM were measured. Transcript abundances were fit with a linear regression model (black line) and a 95% confidence interval (yellow shade). Mixed water layer samples originated from the epipelagic region but could not be classified to the surface or deep chlorophyll maxima. Nitrite concentrations <0.001 µM are not shown. In brackets: KEGG orthologous group; *napA*: periplasmic nitrate reductase; *norB*: nitric oxide reductase subunit B; *nosZ*: nitrous-oxide reductase; *nirS*: nitrite reductase (NO-forming) / hydroxylamine reductase; *nirK*: nitrite reductase (NO-forming). Data adapted from Salazar et al., 2019 [67], see Table 5.

The detection and expression of denitrification genes in the ocean is generally associated with denitrifying bacteria in oxygen-depleted regions. However, here we have shown that an aerobic bacterium, *P. inhibens*, expresses denitrification genes while interacting with an oxygenic photosynthesizing host in co-culture. Many other Roseobacters are known to be aerobes, often in close interactions with photosynthesizing organisms [29, 36, 37, 64, 65]. Yet numerous aerobic Roseobacters continue to carry denitrification genes [31] (Table S1). As a first step towards bridging between our laboratory observations and the marine environment, we examined whether bacterial denitrification genes co-occur with high oxygen and chlorophyll levels in the ocean. Such co-occurrence could indicate the co-existence and potential interaction, of phytoplankton and bacteria with NO-producing potential. We searched the Ocean Gene Atlas (OGA) [66], focusing on sampling points in the deep chlorophyll maximum layer (DCM) where photosynthesizing microalgae thrive. Since the DCM does not necessarily coincide with oxygen maxima levels, we specifically analyzed locations where high levels of oxygen were measured in the DCM (Fig. S6A). We found that the genes *nirK* and *nirS*, both encoding nitrite reductases harbored by members of the Roseobacter group [31], are detected in these regions. In addition, genes encoding the NO reductase subunits, *norC* and *norB*, were found in all locations where *nirK* and *nirS* were detected (Fig. S6B). Moreover, a recent survey of oceanic metatranscriptomes reported on the occurrence of denitrification gene transcripts (*napA*, *nirS*, *norB* and *nosZ*) in oxygenated marine environments [67]. The abundances of the analyzed denitrification gene transcripts exhibited a negative correlation with bulk oxygen concentrations; transcript levels decreased as oxygen concentrations increased (Fig. 5—*napA*, *norB*, *nosZ* and *nirS*). We expanded this analysis to *nirK*, the gene encoding the enzyme that reduces nitrite to NO in *P. inhibens*. We found that *nirK* transcription did not decrease with rising oxygen levels (Fig. 5—*nirK*). Transcript abundances of *nirK* were variable, did not correlate with oxygen concentrations and were frequently high in phytoplankton-rich areas of the ocean (represented by the deep chlorophyll maximum in Fig. 5). Furthermore, in oxygenated locations with high transcript abundances, nitrite levels of 0.1–1.5 µM were measured. These data demonstrate that bacterial *nirK* is expressed in oxygenated ocean waters, that these regions harbor photosynthesizing microorganisms and that nitrite levels in these locations are detectable. It should be noted that the measured nitrite concentration represents the sum of processes that produce and consume nitrite. Therefore, gene expression data rather than actual compound concentration could indicate nitrite-consumption, especially in sites where nitrite levels are under the detection limit. We cannot rule out the possibility that local microaerophilic conditions drive denitrification in environments that are largely oxygenated. However, under microaerophilic conditions that promote denitrification we would expect a similar expression pattern of all denitrification genes. While further studies in the environment are needed to elucidate the mechanism underlying these findings, the link between these observations might be the novel inorganic nitrogen exchange revealed in the current study.

By examining the taxonomic distribution of bacteria in the DCM, we found that a high percentage of bacterial species that were detected in this oxygen-rich region and that carry denitrification genes, are from the *Rhodobacteraceae* family, to which marine Roseobacters belong (Fig. S6C). In addition, we identified metagenome-assembled genomes (MAGs) isolated from the ocean surface that belong to the Roseobacter group [68]. A phylogenetic tree was built using the assembled MAGs, along with genomes of Roseobacters that were previously experimentally validated to be either aerobic (Table S1, and references within) or facultatively anaerobic (Table S4, and references within) (Fig. S6D). It should be noted that these studies (referenced in Tables S1 and S4) did not test microaerophilic conditions, and thus we define bacteria as aerobes or facultative anaerobes according to experimental results in previous studies. Interestingly, the phylogenetic tree revealed that several MAGs grouped together with aerobes, and that these bacteria carry only part of the denitrification genes, but not all the genes essential for the full canonical denitrification pathway (Fig. 1A, Fig. S6D). Therefore, it is possible that like *P. inhibens*, these bacteria use denitrification intermediates for reasons other than bioenergetics. Taken together, the environmental data support a scenario in which bacteria that carry and express denitrification genes co-occur, and possibly interact, with photosynthesizing microorganisms. Therefore, bacterial NO production in oxygen-rich waters might be an overlooked process in interspecies interactions and in the marine nitrogen cycle.

## Discussion

Our study demonstrates a novel route of microbial inorganic communication and highlights the need to expand our view beyond organic metabolic exchange. The data we present suggest that bacterial pathogenicity towards algae involves inorganic nitrogen compounds, independent of the organic routes that were previously described [29, 37, 38, 56, 69–73]. Previous work on the *G. huxleyi*—*P. inhibens* model system revealed that algae exude tryptophan which is converted by bacteria to an algal growth hormone [29]. High levels of the growth hormone triggered algal death. In the previous study, bacteria became pathogens as algae aged, but the mechanism that allowed bacteria to sense algal aging remained elusive. The algal phenylpropanoid *p*-coumaric acid (pCA) has been suggested to act as a senescence signal secreted by *G. huxleyi* [69, 74]. But the function of pCA in the algal cell and in co-cultures is largely unknown. Here, we identify *G. huxleyi* nitrite exudation; a chemical signal that indicates algal growth-phase and promotes bacterial transition to pathogenicity. It is possible that the algal nitrite is a signal that elicits multiple pathogenicity pathways in bacteria, thus offering a novel mechanistic link between algal growth-phase and bacterial pathogenicity. Interestingly, the influence of an algal secreted compound on bacterial denitrification was previously demonstrated [75], underscoring the complex network of algal-bacterial metabolic exchange and its physiological outcomes. Importantly, nitrite secretion was previously documented in various phytoplankton groups, including diatoms and dinoflagellates [33]. The mechanism of nitrite release is not clear, yet it appears to be common and to function for reasons unrelated to bacteria. It is likely that bacteria have evolved to recognize and exploit the algal nitrite. Both pCA and nitrite could be seen as indicators of algal senescence, and therefore highlight a possible bacterial response towards aging algal cells. The various redundant pathways described here and in previous studies [29, 37, 38, 56, 69–73] that enable bacteria to promote death of their algal partner point to the importance of this process in microbial interactions.

It was previously shown that bacteria that harbor partial denitrification pathways are able to use the encoded enzymes for non-denitrifying functions [76–78]. Earlier studies have discussed the occurrence of denitrification genes in bacterial strains that are not capable of producing energy via denitrification. Horizontal gene transfer is a key mechanism for the acquisition of denitrification genes by bacteria [79], including bacteria that do not possess the physiological ability to carry out the denitrification process. Without appropriate physiological context, denitrification genes might not be transcribed, and transcription might not necessarily lead to production of active enzymes [80]. Furthermore, it is possible that certain bacterial strains can produce energy through denitrification under specific conditions, but these conditions have not yet been recapitulated in laboratory experiments. Therefore, we cannot rule out the possibility that *P. inhibens* DSM17395 can use its denitrification capabilities for respiratory functions under conditions that are yet to be tested, such as microaerophilic environments. Our data reveal a novel role of denitrification intermediates during inter-species interactions. In our algal-bacterial model system, the exchange of inorganic nitrogen species allows sensing and killing of a neighboring organism. Aerobic *P. inhibens* bacteria harbor denitrification genes and express them under oxygen-rich conditions for reasons unrelated to bioenergetics (Fig. 2, Fig. S2), and mutant bacteria that do not carry the denitrification genes are impaired in their ability to promote death in the algal partner. Rather than being an ephemeral intermediate in respiration-related denitrification, NO appears to play a role in the algal-bacterial interaction and is involved in algal death. Yet, it is difficult to determine which selection pressure caused the maintenance of denitrification genes in *P. inhibens*. Killing an aging algal host provides a boost of nutrients that promotes bacterial growth. However, the occurrence of denitrification genes was shown to be widely distributed, and not directly associated with present-day environmental selection [81]. Therefore, while it is advantageous for bacteria to promote death of aging algae through denitrification intermediates, this is not necessarily the selection pressure that favored maintenance of denitrification genes in aerobic bacteria.

Data from the current work and from previous studies demonstrate that NO can act as a signaling molecule in various algae [18, 47–49, 82]. In diatoms, NO levels were shown to determine cell fate [48, 49]. Exposure to lethal doses of specific aldehydes resulted in high intracellular NO levels followed by cell death. But NO was also generated as a signaling molecule after exposure to sublethal stress levels. Owing to the diffusible nature of this small gaseous molecule, NO diffused between adjacent diatoms thereby propagating the danger signal in the population and inducing resistance. Interestingly, treating diatoms with sub-lethal doses of a stress-inducing aldehyde conferred resistance to high levels of the same aldehyde. In our system, however, pre-treatment with sub-lethal NO levels had the opposite effect and algae became susceptible to lower levels of NO (Fig. S7). Similarly, Murik and Kaplan [83] showed that exposure to sublethal doses of hydrogen peroxide (H_2_O_2_) results in hypersensitivity to subsequent exposure to H_2_O_2_. These results highlight the similarities in the algal response towards reactive oxygen species (ROS) and reactive nitrogen species (RNS).

During early viral infection of *G. huxleyi*, intracellular NO was suggested to serve as an antioxidant. High levels of intra- and extra-cellular NO were measured post-infection when algae lyse, both in the lab and at sea. These previous studies provided significant insights into the ecophysiology of NO signaling and antioxidant activity in phytoplankton in response to external stimuli such as viral infection [18, 47] or specific aldehydes [48, 49].

**Fig. 6:**
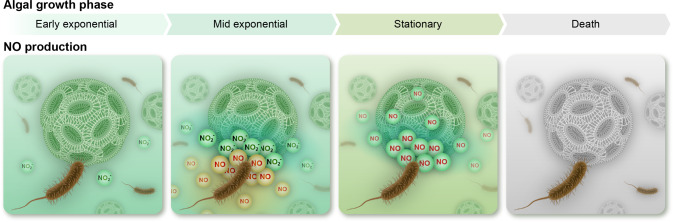
A model depicting inorganic nitrogen exchange during algal-bacterial interactions. Inorganic nitrogen exchange underlies the algal-bacterial dynamic interaction. Algae secrete nitrite in a growth-phase dependent manner, with a nitrite peak during mid exponential growth. Bacteria reduce algal-secreted nitrite to NO via denitrification. NO secreted by bacteria triggers algal death at stationary phase, during which algae produce NO. Algal-secreted NO propagates the death signal among algal cells, causing the collapse of the algal population.

In the algal-bacterial interaction, NO appears to be central both in executing algal death (in a yet unknown molecular mechanism) and in propagating the signal among the algal population (Movie S1). Our observations add complexity to previous reports on the role of NO in microalgae. We expand current knowledge by demonstrating that NO from a bacterial source can trigger algal NO production and propagation in the population (Fig. 6). When the NO signal spreads in the population it culminates in an orchestrated PCD that releases a burst of nutrients. As bacterial NO production is elicited by algal nitrite secretion, this assures the proper timing of algal death- when algae are no longer growing exponentially. It remains to be resolved whether NO itself - from either algal or bacterial origin- is the lethal molecule or whether it triggers a cellular cascade that leads to cell death. In light of the current study, bacteria might be a source of NO in non-axenic algal cultures (i.e. cultures containing bacteria) and in algal blooms. Bacterial NO would influence algal physiology and impact NO signaling during interactions between algae, their viruses and their grazers.

The role of NO as an extracellular signaling molecule is possible due to the close proximity between interacting algae and bacteria [29]. Bacterial NO can diffuse into algal cells, efficiently acting as a signal that triggers algal death [13]. In multicellular systems, local NO concentrations are crucial in determining specific intracellular responses of the affected cell [84, 85]. Similarly, the differential expression of denitrification genes in bacteria is regulated by nanomolar changes in extracellular NO concentrations [86]. In *G. huxleyi*, exposure to low NO concentrations increased population susceptibility to reactive oxygen species [18]. Thus, the local concentration of NO in the algal phycosphere, the immediate volume surrounding the algal cell, might greatly influence algal fate and the nearby microbial population. Of note, compound transport in the algal phycosphere is entirely diffusive due to the thin boundary layer surrounding microorganisms [87]. Therefore, concentrations of NO (and nitrite too) are expected to greatly differ between the phycosphere and the bulk liquid. Marine microbial “hot-spots”, such as marine snow particles and algal blooms, promote the development of local conditions with restricted diffusion, resulting in high concentrations of secreted compounds [88–91], which are otherwise diluted in the open ocean [92]. Thus, short-lived, diffusible signals like NO might stimulate various localized responses, and are expected to differ between bloom and non-bloom conditions. Previous studies demonstrated that PCD in microorganisms is triggered by different stimuli that initiate a cascade of intracellular signaling events, eventually resulting in cell death. The cellular response is affected by the type, intensity and duration of the stimulus [11, 93]. Thus, PCD in microorganisms is dose dependent. In the current study, both bacterial density and consequently bacterial NO increase during the time between the peak of algal nitrite and the detection of algal demise. It is currently unknown what are the local NO concentrations that are needed to trigger algal PCD, and when are these conditions met in the algal-bacterial interactions. The study of metabolic exchange that occurs in the algal phycosphere is currently limited by the lack of technology for direct probing of local processes. Accurately determining levels of key compounds and gases like oxygen and NO at the interface between individual algal and bacterial cells is crucial for establishing the pertinent concentrations that cells encounter. Ingenuity and development of technological capabilities would greatly benefit this growing field of studies.

Data in our study indicate that aerobic bacteria can express denitrification genes for purposes other than respiration and energy production. This phenomenon appears of ecological importance beyond the *G. huxleyi – P. inhibens* interaction. We were able to detect denitrification genes and transcripts in oxygen-rich regions (Figs. 5, S6). We cannot rule out the possibility that the detected genes and transcripts belong to facultative anaerobes such as *P. inhibens* T5, a closely related species to our model bacterium. However, analysis of genomes of known aerobic bacteria revealed that they harbor subsets of denitrification genes (Table S1). This is contrary to facultative anaerobes such as *P. inhibens* T5, which harbors additional denitrification genes enabling anaerobic growth (Table S4) [46]. A literature survey revealed that expression of denitrification genes and the occurrence of active denitrification has been previously reported in oxic conditions both in culture experiments and in environmental studies [94–100], but the underlying ecophysiology was not discussed. As microbial NO can act across kingdoms, it might have overlooked roles in microbial ecology. The prevalence and environmental significance of microbial denitrification in oxygen-rich environments remains to be determined.

## Materials and methods

### Strains and general growth conditions

The bacterial strain of *Phaeobacter inhibens* DSM 17395 was purchased from the German collection of microorganisms and cell cultures (DSMZ, Braunschweig, Germany). The bacterial Δ262 plasmid cured mutant was kindly provided by the lab of Jӧrn Petersen, Leibniz Institut DSMZ, Germany [45]. Bacteria were plated on ½ YTSS agar plates containing 2 g yeast extract, 1.25 g tryptone and 20 g sea salt per liter (all purchased from Sigma-Aldrich, St. Louis, MO, USA). Pure bacterial cultures were grown in CNPS medium consisting of L1-Si medium (see below) supplemented with glucose 5.5 mM, Na_2_SO_4_ 33 mM, NH_4_Cl 5 mM, KH_2_PO_4_ 2 mM, (all purchased from Sigma-Aldrich) [29, 101]. Cultures were incubated at 30 °C shaking at 130 rpm.

The axenic algal strain of *Gephyrocapsa huxleyi* CCMP3266 was purchased from the National Center for Marine Algae and Microbiota (Bigelow Laboratory for Ocean Sciences, Maine, USA). Algae were grown in L1 medium according to Guillard and Hargraves [102], with the exception that Na_2_SiO_3_ was omitted following the cultivation recommendations for this algal strain, and the medium was referred to as L1-Si. Algae were grown in standing cultures in a growth room at 18 °C under a light/dark cycle of 16/8 h. Illumination intensity during the light period was 150 mmoles/m^2^/s. Absence of bacteria in axenic algal cultures was monitored periodically both by plating on ½ YTSS plates and under the microscope.

Co-cultures of *G. huxleyi* and *P. inhibens* were prepared as follows: algal cell concentrations from a late exponential phase culture were counted using a hemocytometer in a fluorescent microscope. An inoculum of 10^4^ algal cells was introduced into 30 ml of L1-Si medium and incubated as described above. After four days of algal growth, *P. inhibens* biomass from a plate was resuspended in 0.5 ml L1-Si, diluted X10^4^ and 20 µl were added to 30 ml of algal culture, resulting in 10–100 colony forming units (CFU) per ml. The co-cultures were incubated in a growth room under the conditions described above for algal cultures. Sampling days are indicated as days following bacteria addition.

### Genetic manipulation of *P. inhibens* bacteria

To delete the *nirK* gene, the mutant Δ*nirK* (strain ES90) was generated as follows: three fragments were PCR amplified; a region of ~900 bp upstream of *nirK* was amplified using primers 1 and 2 (Table 1), a gentamicin resistance cassette was amplified from plasmid pMS1 (Table 2) using primers 3 and 4 and a region of ~900 bp downstream region of *nirK* was amplified using primers 5 and 6. After fragments amplification, the fragments were joined using NEBuilder HiFi DNA assembly kit (New Englands BioLabs) following the manufacturer instructions. The resulting assembled product was further amplified using primers 7 and 8 which left T/A overhangs needed for further integration into the pCRII-TOPO vector (Invitrogen) following the manufacturer instructions. The resulting plasmid (pAA10, Table 2) was electroporated into *P. inhibens* using 10 μg of DNA for each 300 μl aliquot of electrocompetent cells. Preparation of competent cells and transformation was performed as previously described [103]. Cells were transformed and then plated on 1/2YTSS plates containing 30 μg/ml gentamycin and resistant colonies were verified using PCR and sequencing.

**Table 1:** Primers used for genetic manipulation in this study.

Primer number	Primer name	Sequence
1	nirK 5F1	CTTATGGATCACTGAGGAGC
2	nirK MFR-5R	TTTGTTCAGCGGCTTGTAGTCGCTCTCCTTGTCTTAGG
3	gent MF	TACAAGCCGCTGAACAAA
4	gent MR	GAAACCAAGCCAACCAGG
5	nirK 3R1	GCACCTTATCCAACACGC
6	nirK MRR-3F	CCTGGTTGGCTTGGTTTCGACCATAGGAGAGCACAATG
7	nirK 5F2	GCAATGTGGTACCTGTGAG
8	nirK 3R2	CGGATTAAGGCTAAAGTGGA
9	norD 5F1	GCCCCAACAACACTAGAAC
10	norD MFR-5R	TTTGTTCAGCGGCTTGTACATGCGTCACATCTTATCG
11	norC 3R1	GCGGAGACATTCTATTTGC
12	norC MRR-3F	CCTGGTTGGCTTGGTTTCGTTGATCGCTTGGTGATAG
13	norC 3R2	CGGACAGCTATTTGGAATTG
14	norD 5 F2	GATAACCTCACGCAGGATTAC
15	nnrS-5F	GTACAAAAAAGCAGGCTCCGAATTCGCCCTTACCGCTGACACCTAGCATCAGATCGC
16	nnrS-5R	GCATTACAGTTTACGAACCGAACAGGCTTATGTCAACACGTTGTCAGAGCCGAGACCCG
17	nirV-3F	GCCTTCTATCGCCTTCTTGACGAGTTCTTCTGACCTTAATCCGGGCGCACCGC
18	nirV-3R	CTTTGTACAAGAAAGCTGGGTCGAATTCGCCCTCCACGCCAATCTGAGCAATCCG
19	F-PC-Km	TTGACATAAGCCTGTTCGGTTCG
20	R-Km	TCAGAAGAACTCGTCAAGAAGGCG

**Table 2:** Plasmids used in this study.

Plasmid name	Origin	Comments
pMS1	Gift from Dr. Mo Seyedsayamdost, Princeton University, USA	Constructed using the pBluescript vector and used in the current study for amplifying the gentamycin resistance cassette
pYDR1	Lab collection	Constructed using pBBR1MCS-5 and used in the current study for amplifying the kanamycin resistance cassette
pAA10	Current study	Used in the current study to replace *nirK* with a gentamycin resistance cassette
pAA12	Current study	Used in the current study to replace the *nor* operon from *norC* to *norD* with a gentamycin resistance cassette
pDN6	Current study	Used in the current study to replace region *nnrS* to *nirV* with a kanamycin resistance cassette

To delete the *nor* operon, the mutant Δ*nor* (strain ES89) was generated as follows: ~1000 bp regions upstream of the *norC* gene and downstream of the *norD* gene were PCR-amplified using the primers 9, 10, 11 and 12, respectively (Table 1). The gentamycin resistance cassette was amplified from plasmid pMS1 using primers 3 and 4. After fragments amplification, the fragments were joined using NEBuilder HiFi DNA assembly kit (New Englands BioLabs) following the manufacturer instructions. The resulting assembled product was further amplified using primers 13 and 14 which left T/A overhangs needed for further integration into the pCR8/GW/TOPO vector (Invitrogen) following the manufacturer instructions. The resulting plasmid (pAA12, Table 2) was introduced into *P. inhibens* by electroporation using 10 μg of DNA for each 300 μl aliquot of electrocompetent cells. Cells were transformed and then plated on 1/2YTSS plates containing 30 μg/ml gentamycin and resistant colonies were validated by both PCR and DNA sequencing.

To delete all denitrification genes on the native 262 kb plasmid of *P. inhibens*, the mutant Δ*nnrS*→*nirV* (strain ES121) was generated as follows: ~1000 bp regions upstream of the *nnrS* gene and downstream of the *nirV* gene were PCR-amplified using the primers 15, 16, 17 and 18, respectively (Table 1). The kanamycin resistance cassette was amplified from plasmid pYDR1 (Table 2) using primers 19 and 20. The amplified fragments were assembled and cloned into the pCR8/GW/TOPO vector (Invitrogen) using restriction-free cloning [104]. The resulting plasmid (pDN6, Table 2) was introduced into *P. inhibens* by electroporation using 10 μg of DNA for each 300 μl aliquot of electrocompetent cells. Cells were transformed and then plated on 1/2YTSS plates containing 150 μg/ml kanamycin and resistant colonies were validated by both PCR and DNA sequencing.

### Chemical treatments

When indicated, cultures were treated with the NO donor DEANO (Diethylammonium (Z)-1-(N,N-diethylamino)diazen-1-ium-1,2-diolate, Cayman chemical, Ann Arbor, MI, USA). Stock solutions of DEANO were prepared by dissolving the chemical in ultrapure water followed by immediate freezing at -80 °C in aliquots. To test the stability of the compound, every week a freshly thawed aliquot was added to algae in a concentration of 80 µM (the minimum inhibitory concentration, Fig. S7). We found that the compound is stable in water for at least 3 months. After three months, the stock was discarded, and a new stock was prepared. The concentration of DEANO used in each experiment is detailed in each corresponding figure legend. The NO scavenger carboxy-PTIO (c-PTIO, ThermoFisher, Waltham, MA, USA) was diluted in ultrapure water and stored in -20 °C in aliquots. The chemical was added to a final concentration of 20 µM when indicated.

### Monitoring algal growth in cultures

Algal growth in cultures was monitored by a CellStream CS-100496 flow cytometer (Merck, Darmstadt, Germany), using 561 nm excitation and 702 nm emission. For each sample 50,000 events were recorded.

### Monitoring bacterial growth in co-cultures

Bacterial growth in co-cultures was evaluated by sampling co-cultures at different time points, as indicated. Samples were serially diluted and plated on ½ YTSS plates. CFUs were counted and the concentration in the sampled culture was calculated. CFUs may include rosettes as well as individual bacteria.

### Monitoring bacterial growth in pure cultures

Bacteria were grown in CNPS as described above. Cultures were monitored daily by OD_600_ measurements in an Ultrospec 2100 pro spectrophotometer (Biochrom, Cambridge, UK) using plastic cuvettes. Cell numbers were calculated based on OD_600_ values.

### Inorganic species measurements

Axenic algal cultures were grown as previously described. On the day of sampling, 1 ml of culture was filtered through a 0.1 µm syringe filter in three biological replicates. The filtrates were then analyzed for their inorganic species concentrations using Thermo Scientific Gallery Plus discrete auto analyzer (ThermoFisher). The inorganic species that were measured were NO_3_^−^, NO_2_^−^, PO_4_^3−^, SO_4_^2−^ and NH_4_^+^ using the manufacturer instructions. Briefly, the retrieved values represent the contribution of a given element (such as N) to the entire molecule (such as NO_3_^−^). The retrieved values are therefore annotated N-NO_3_^−^, N-NO_2_^−^, P-PO_4_^3−^, S-SO_4_^2−^ and N-NH_4_^+^. To calculate molar concentrations of the molecule, two conversions were conducted:

1. Conversion from element contribution to the entire molecule concentration:
  $$X_{mg/L(molecule)} = X_{mg/L(element)} \ast Conversion\;factor$$

$$X_{mg/L(molecule)}-{{{{{{{\mathrm{Molecule}}}}}}}}\;{{{{{{{\mathrm{concentration}}}}}}}}$$

$$X_{mg/L(element)}-{{{{{{{\mathrm{Element}}}}}}}}\;{{{{{{{\mathrm{concentration}}}}}}}}$$

$$Conversion\;factor - \frac{{Molecule\;molar\;mass}}{{Element\;molar\;mass}}$$

(2) Conversion from mg/L units to micro molar units:
  $$\frac{{X_{mg/L(molecule)}}}{{Molecule\;molar\;mass}} \ast 10^{{{{{{{\mathrm{3}}}}}}}}$$

L1-Si growth medium and filtered seawater were analyzed as controls. Certified ion standards were provided by the manufacturer with a standard concentration deviation of ±0.7% for all ions and ±0.4% for ammonium. Standards were used for calibration.

### Extracellular nitrite measurements

Extracellular nitrite was monitored using the Griess assay as follows: cultures were sampled on indicated days and filtered through a 0.2 µm syringe filter. Fresh standard curves were constructed using known concentration of NaNO_2_ (Sigma-Aldrich). Samples and standards were diluted 1:1 using Griess reagent (Sigma-Aldrich), according to the protocol of the manufacturer, in a 96 well plate and incubated for 15 min in the dark. Samples were measured in triplicates at 545 nm using an Infinite M Plex plate reader (Tecan, Männedorf, Switzerland).

### Anaerobic growth

A serum bottle was filled with 100 ml filtered seawater and supplemented with 0.25 mg/L resazurin and 10 mM NaNO_2_. The medium was brought to boiling on a magnetic stirrer plate and then supplemented with 1.5 ml cysteine-HCl (1 mg/ml) solution (freshly prepared and titrated to pH=8.1). Next the bottle was purged with nitrogen until the color of the medium became transparent. The bottles were sealed and sterilized by autoclaving.

The following steps were conducted in a Coy Lab Products anaerobic chamber. After sterilization, the medium was supplemented with glucose (5.5 mM), Na_2_SO_4_ (33 mM), NH_4_Cl (5 mM) and KH_2_PO_4_ (2 mM) using a syringe with a needle. Next, the medium was divided into six 20 ml sterile and sealed serum bottles (10 ml medium was added to each bottle). Finally, the medium was inoculated with bacteria in triplicates. For inoculation, the strains *P. inhibens* DSM 17395 and *P. inhibens* DSM 16493 (T5) were grown in oxic conditions in CNPS medium (as described in section “Strains and general growth conditions”) for 24 h. The strains were inoculated into the serum bottles to a final OD_600_ of 0.01 using a syringe with a needle. The serum bottles were incubated at 30 °C in the anaerobic chamber. The OD_600_ of the samples was monitored along 29 days.

### Quantitative real time PCR (qPCR)

To test the expression of *nirK* and *norB* genes in pure bacterial cultures, bacteria were grown for 48 hours in CNPS as detailed above. Cultures were treated with 10 µM nitrite for the indicated times after which cells were harvested for RNA extraction. To test the expression of the *nirK* gene in algal-bacterial co-cultures, the cultures were grown as detailed above for the indicated times and cells were harvested for RNA extraction. Importantly, RNA was extracted from both algae and bacteria. Therefore, primer specificity was assessed using pure algal and bacterial cultures to validate that primers detect only bacterial genes (Table 3). Finally, to test the expression of genes that are known hallmarks of algal PCD, pure algal cultures were grown for 48 hours as indicated above (Table 4). These cultures were then treated with 100 µM of the NO-donor DEANO for 18 hours prior to harvest.

**Table 3:** qPCR primers for bacterial genes.

Gene name	Accession number	Forward Primer	Reverse Primer
*recA*	AFO91236.1	GCTGACACCCAAGTCGGAG	AGCCGAACATAACGCCAATCT
*gyrA*	AFO91225.1	GCCGATTCCTGACCTCCTTC	TCAGCTTATGTCGGGCTTCG
*nirK*	AFO93407.1	CGGACAGTCAGATGGAACAC	ATTCATTCCGTGGGTGACAT
*norB*	AFO93403.1	TGTTGACCGAGAAGTGGTGG	TTCCAGACCATCACGAAGGC

**Table 4:** qPCR primers for algal genes.

Annotated gene product	Accession number	Forward Primer	Reverse Primer
β tubulin	XM_005764044.1	CAACATGAAGTGCGCCATCT	CCTCGGTGAACTCCATCTCG
Ribosomal protein L13	XM_005781721.1	ACCAGCACTTCCACAAGACG	TGCCGCAGCTTGTAGTTGTA
Glutathione synthetase (GSHS3)	XM_005760150.1	CTCCGGCAGGTCGAGCTAAA	GAGGCGTGCATGTCTTGCAG
Putative L-ascorbate peroxidase	XM_005784352.1	CGTGTCGACGCCTTAACAG	CACGATAGCCGGATGAGAAT
Autophagy-related protein 8C-like	GIZZ01018991.1	GGGCAGTTCGTGTACGTGAT	CTCGAGCTCTCCGAATGTGT
Thioredoxin f	GIZZ01024661.1	CACCAAGGCTGAGTTTGACA	GTAGAACTGGAAGGTCGGC
Putative metacaspase	GIZZ01008715.1	CCGACCACCAACTTCAACT	GGCTTTTCGTGGTAGTTGTC
Nitric oxide synthase 2	MZ773650	GTGGGTCTCGGTCGGATG	AGCCACCCCTGCTCCTAC

For RNA extraction, 10^6^ algal cells or 10^8^ bacterial cells were harvested by centrifugation at 4000 rpm for 10 min. RNA was extracted using the Isolate II RNA mini kit (Meridian Bioscience, London, UK) according to the manufacturer instructions. Cells were ruptured in RLY buffer containing 1% β-mercapto-ethanol by bead beating with 100 µm low binding silica beads (SPEX, Metuchen, Netherland) for 5 min at 30 mHz. Approximately 1.4 µg of DNA was treated with 4 µl Turbo DNAse (ThermoFisher), in a 50 µl reaction volume. RNA samples were cleaned and concentrated using RNA Clean & Concentrator-5 kit (Zymo Research, Irvine, CA, USA) according to the manufacturer instructions. Algal-bacterial RNA samples were cleaned of algal ribosomal RNA using *G. huxleyi* riboPOOL kit (siTOOLs Biotech GmbH, Planegg, Germany) according to the manufacturer instructions, followed by additional RNA cleaning as described above. Equal concentrations of RNA were utilized for cDNA synthesis using Superscript IV (ThermoFisher), according to manufacturer instructions. qPCR was conducted in 384 well plates, using SensiFAST SYBR Lo-ROX Kit (Meridian Bioscience) in a the QuantStudio 5 (384-well plate) qPCR cycler (Applied Biosystems, Foster City, CA, USA). The qPCR program ran according to enzyme requirements for 40 cycles. Results were analyzed using a relative standard curve using the QuantStudio 5 software. Primer efficiencies were determined by qPCR amplification of serially diluted cDNA. Only primer pairs with a minimum of 80% efficiency were selected, with the exception of primers for Thioredoxin-encoding gene which exhibited 65% efficiency. Samples were normalized using two housekeeping genes: bacterial housekeeping genes- *recA* and *gyrA* (Table 3) and algal housekeeping genes—*β-tubulin* and *rpl13* (Table 4). DNA contamination was not detected when applying the same program on RNA samples that were not reverse-transcribed. Relative gene expression levels were compared to non-treated samples grown under the same conditions. In co-cultures, relative gene expression levels in bacteria at the indicated time points were compared to expression levels on day 10.

### Light microscopy

Fluorescence and phase contrast images were obtained using a Nikon Eclipse Ti2-E inverted microscope equipped with a CFI Plan Apochromat DM 100X objective lens (Nikon, Tokyo, Japan). All samples were spotted on thin 1% agarose pads for visualization at room temperature. Images were acquired using an Andor Zyla 4.2 camera controlled with Nis elements software. The 470/24 and 575/25 Lumencor Spectra X Chroma excitation filters were used. DAF-FM was captured using an ET519/26 m filter. Images were processed identically for compared image sets.

Movie S1 was generated by imaging five random fields. For each field, an image was captured every 3 minutes, for a duration of 1 h. At each time point, the focus was adjusted manually and the field was imaged using a phase contrast lens and subsequently using a ET519/26 m filter to image the fluorescent signal of DAF-FM.

### DAF-FM staining for microscopy

For intracellular detection of NO in bacteria, bacterial strains were grown for 24 h from an initial OD_600_ of 0.04, in CNPS medium under the conditions described above. For the last 2 h, bacteria were incubated with or without 100 µM NaNO_2_. Cells were then centrifuged in 8000 rpm for 5 min at room temperature, resuspended in 10 µM DAF-FM Diacetate (4-Amino-5-Methylamino-2’,7’-Difluorofluorescein Diacetate) (ThermoFisher) diluted in L1-Si, and incubated in the dark for 30 min. Cells were washed 3 times in L1-Si prior to imaging by fluorescent microscopy on agarose pads as described above. Image background was subtracted from images of bacterial cells which were not incubated with DAF-FM.

In order to detect the propagation of NO among algal cells, 1 ml of a late exponential phase algal culture was treated with 100 µM DEANO and incubated for 8 h. The treated algal culture was then thoroughly washed 3 times with seawater to remove residuals of the chemical treatment. In parallel, 1 ml of an exponential phase algal culture was stained with DAF-FM as described above. The DAF-FM stained algae were seeded with DEANO-treated algae in a ratio of 4:1 respectively. The mixed culture was then incubated for 30 min at room temperature in the dark, mounted on 1% agarose pads, and monitored under the microscope as described above. As a control, the same experimental procedure was used on algal cultures only without the addition of DEANO. No NO fluorescence signal was detected in control samples.

### Extracellular NO measurements

Extracellular nitric oxide in bacterial and algal cultures was measured by Liposome-Encapsulated-Spin-Trap (LEST) and electron paramagnetic resonance (EPR) spectroscopy, based on the method previously published by Hirsh, et al. [47]. with several adjustments. Multi Laminar Vesicles (MLVs) were prepared from a 9:1 molar ratio of the phospholipids POPC and DPPG (Avanti Polar Lipids, Alabaster, AL, USA) in chloroform containing 2% methanol and 1% ultrapure water. The phospholipid mix was divided into glass bottles, evaporated under nitrogen flow and lyophilized overnight. Bottles were then capped and kept in –20 °C for further use. LESTs were prepared one day prior to incubation with cells. On the day of preparation, the lipid film was dissolved in 1 ml buffer solution per 100 mg phospholipid of MLV Prep Buffer containing 10 mM MES pH 6.4 (Sigma-Aldrich) and 50 mM NaCl, 10 mM of the spin trap MGD (Santa Cruz Biotechnology, Heidelberg, Germany) and 2 mM ammonium iron (II) sulfate (Sigma-Aldrich). Glass bottles were capped with a rubber septum and the head space was flushed with nitrogen for 10 min. The lipid film was then completely suspended in the MLV Prep Buffer. Lipids were then frozen in liquid nitrogen and thawed in room temperature water five times to create frozen-and-thawed MLVs (FAT-MLVs). The rubber cap was then removed and EDTA at pH=6.4 (Sigma-Aldrich) was added to a final concentration of 2 mM. MLVs were then diluted 1:2 in NO Assay Buffer containing 20 mM HEPES at pH=7.4 (Sigma-Aldrich) and 140 mM NaCl, and centrifuged at 20,000 g for 30 min at 4 °C. The supernatant was discarded and the MLVs were resuspended in NO Assay Buffer to a final volume of 2.5 ml. MLVs were loaded on a pre-equilibrated PD-10 de-salting column (GE Healthcare, Chicago, IL, USA) and collected with NO assay buffer in two 1.5 eppendorf tubes, followed by a second centrifugation at 20,000 × *g* for 30 min at 4 °C. MLVs were then resuspended at 480 µl volume per 100 mg phospholipid, and kept overnight at 4 °C. Axenic algal cultures or algal-bacterial co-cultures were grown as described above. Bacterial cells were grown for 24 hours from an initial OD_600_ of 0.04, in CNPS medium under the conditions described above, to a concentration of 10^7^ cells/ml. On the day of measurement, bacterial and algal cultures were centrifuged at 4000 rpm and resuspended in 1 ml or 5 ml of growth medium, respectively. Then, 30 µl of pre-made LESTs were added to each sample, as well as NaNO_2_ when indicated.Samples were incubated in the dark for 5 h. During incubation, NO secreted by cells was absorbed by LESTs and stabilized by the MGD molecule in the LESTs. Following incubation, samples were centrifuged at 20,000 × *g* for 30 min at 4 °C, resuspended in 100 µl, flash frozen in liquid nitrogen and kept at -80 °C for a maximum of 7 days until measured by electro-paramagnetic resonance (EPR). In each experiment, a sample treated with the NO-donor DEANO was used as a positive control to verify proper LEST preparation and NO detection. As negative control, LESTs were incubated with nitrite, and no NO was detected in these samples.

For EPR measurements samples were thawed and drawn up into microcapillary tubes. EPR spectra were recorded on a Bruker ELEXSYS E500 X-band spectrometer equipped with a Bruker ER4102ST resonator at room temperature. Experimental conditions were: 512 points, with microwave power of 20 mW, 0.1 mT modulation amplitude and 100 kHz modulation frequency. Sweep range was 10 mT. NO concentrations were calculated according to a standard curve of known concentrations of Carboxy-proxyl diluted in ultrapure water and measured directly by EPR, without incubation with LESTs. A blank measured using an empty capillary was omitted from the measured values.

### DAF-FM measurements in algae using flow cytometry

One ml of an algal culture at late exponential growth phase was centrifuged at 8000 rpm for 5 min and stained with DAF-FM as detailed above. Cells were then washed with L1-Si and incubated for 30 min in the dark. Cells were washed again, resuspended in 400 µl of L1-Si and divided to aliquots of 50 µl. Each aliquot was diluted with 450 µl L1-Si as control or with 450 µl of bacteria, which were grown for 48 h in CNPS as detailed above. Where indicated, bacteria were killed by treatment with gentamycin (100 µg/ml) and kanamycin (200 µg/ml) for 4 h. Bacterial death was validated by plating a sample of the antibiotic-treated bacteria on 1/2YTSS plates. 10 µM NaNO_2_ was added where indicated. As a positive control for DAF-FM staining, stained algae were treated with 300 µM of the NO-donor DEANO. Samples were incubated in the dark for 2 h and then measured by flow cytometry. Detection of algal cells was conducted as described above. 10,000 algal events were collected. Detected algal events were plotted for DAF-FM fluorescence, using the 488 nm excitation and 528 nm emission filter. Events of high DAF-FM fluorescence were gated according to the control of DEANO-treated algae. The relative percent of gated events was normalized to the background fluorescence of DAF-FM-stained algae with or without NaNO_2_ treatment.

### RNA sequencing

To identify PCD-related genes in *G. huxleyi* CCMP3266, we re-analyzed previously generated transcriptome data [57]. In the previous work, we constructed a transcriptome of *G. huxleyi* CCMP3266 by integrating Illumina total RNA short-reads and PacBio full-length cDNA long-reads into a de novo assembled hybrid transcriptome (accession number: GIZZ00000000). In the same work, we collapsed redundant transcript isoforms into less redundant CCMP3266 gene loci, resulting in a “synthetic genome”, which we used as reference for genetic analyses. In the current study, we used a text search to screen the *G. huxleyi* CCMP3266 transcript-based gene annotation table (Data S2 in [57], Table 5) for functions known to be hallmarks for PCD and oxidative stress. We further filtered the identified, putative PCD and oxidative stress genes for those that were differentially expressed (DE) during four different life phases of the alga in co-cultures with bacteria. The temporal DE analysis was conducted as previously described [57], using the Illumina total RNA sequencing data and the “synthetic genome” of CCMP3266 as reference, but by applying a less stringent adjusted p value cutoff of 0.1. The newly identified, putative *G. huxleyi* CCMP3266 PCD and oxidative stress genes, as well as their temporal expression during the transition from algal growth to demise, is presented as heatmap. The heatmap was generated with pheatmap [105], and rows were hierarchically clustered according to expression patterns with default options.

**Table 5:** Data availability.

Data set	Reference	Link or accession
RNA sequencing	Sperfeld et al. 2022 [57] Data S2	https://zenodo.org/record/5702921
Environmental data	Salazar et al. 2019 [67] Supplemental file: ‘TARATranscriptAbundances.tsv’	https://zenodo.org/record/3539258
Metagenome-assembled genomes (MAGs)	Delmont et al. 2018 [68]	NCBI accession: PRJNA326480

### Bioinformatical identification of algal *noa* genes

The *noa* genes were predicted from the *G. huxleyi* genome sequence [106], and defined based on Illumina short read sequence [107, 108], as described in Feldmesser et al. [109]. Briefly, the sequences used for the initial searches were from *Phaeodactylum tricornitum* (a manually 5’ extended version of Phatr3_J40200, the version 3 accession number of Phatr2_56150 and Phatr2_37004 or v3: Phatr3_EG00845.t1), Arabidopsis (NP_190329.2) and human (NP_000611.1, NP_000616.3, NP_000594.2). The protein sequences containing the YqeH domain, a conserved domain found in NO producing enzymes [49], were used for database searches (BLASTp), multiple alignments (ClustalW 2.1) and phylogenetic trees (Neighbor-Joining in ClustalW, PhyML 3.0) [110, 111] to ensure their belonging to the correct family. *noa* sequences were aligned in MAFFT with the --leavegappyregion and --auto parameters [112]. IQTree 1.6.12 was used to perform maximum-likelihood phylogenetic analyses with the best-fit model (LG + F + R5) according to the Bayesian information criterion and 100 bootstraps [113]. The phylogenetic tree was visualized and annotated in FigTree, and branches were colored according to bootstrap values.

### Environmental data

#### Metagenomic analyses

Environmental data was obtained using the Ocean Gene Atlas (OGA) server [66]. The protein sequences of *nirK* (WP_014881756.1), *norB* (WP_014881752.1) and *norC* (WP_014881753.1) from *P. inhibens* DSM 17395 and *nirS* from *Roseobacter denitrificans* OCh114 (WP_044032999.1) were used as queries. Data analysis was limited to sampling points within the Deep Chlorophyll Maxima (DCM) region in which oxygen and chlorophyll measurements were available. Chlorophyll measurements were plotted against oxygen level measurements for each sampling point. Phylogenetic data was extracted from the taxonomic composition of each sampling point using Krona [114]. The sampling point with the highest number of detected sequences (hits) in the DCM was chosen for data presentation. In the chosen sampling point, the numbers of hits for each gene were as follows: *nirK* 16, *norB* 50, *norC* 22.

#### Transcript abundances of denitrification genes

Transcript abundances of denitrification genes were recently reported by Salazar et al. 2019 [67], as part of the TARA Oceans consortium. Briefly, the consortium sampled prokaryote-enriched RNA from different layers of the ocean and conducted paired-end sequencing on the HiSeq2000 system (Illumina). Sequencing reads were quality-filtered, trimmed and mapped to the functionally annotated Ocean Microbial Reference Gene Catalog v2. Read counts were normalized by gene length, summed-up by KEGG clusters of orthologues groups (KO), divided by the abundance of constitutively expressed marker gene transcripts, and variance-stabilized with DESeq2. The resulting log_2_-transformed transcript abundances represent relative transcript numbers per cell, and are publicly available (https://www.ocean-microbiome.org/). We plotted the data with nitrite and oxygen concentrations (10.5281/zenodo.3473199) in ggplot2, and fitted transcript abundances with the default linear model (lm). The input data used for this analysis is provided in the supplemental materials (TARATranscriptAbundances.tsv, Table 5).

### MAG and Roseobacter tree methods

Metagenome-assembled genomes (MAGs) from the TARA Oceans Project were assembled in Delmont et al. 2018 [68] and downloaded (NCBI accession: PRJNA326480, Table 5). MAGs grouping within the Rhodobacteraceae clade were retained for this analysis. Complete genomes from cultured anaerobes and aerobes in the Roseobacter clade were downloaded and both high-quality MAGs (over 70% complete and less than 10% contaminated) and cultured genomes were annotated using the PROKKA annotation softw0are [115]. Custom hidden Markov models for enzymes encoded by the phylogenetic gene *rpoB* or the denitrification genes *narG, nirS, nirK, norB* and *nosZ* were searched against the annotated genomes to further verify annotations or find genes missed by the automated process in PROKKA. *rpoB* sequences belonging to each MAG or cultured genome were extracted and aligned in MAFFT with the L-INS-i method and --leavegappyregion parameter [112]. IQTree 1.6.12 was used to perform maximum-likelihood phylogenetic analyses on the *rpoB* alignment with the best-fit model (LG + F + I + G4) according to the Bayesian information criterion and 100 bootstraps [113]. The resulting tree was visualized in iTOL and annotated with the presence or absence of each denitrification gene within each genome.

## Supplementary information

Movie S1

Supplemental Material

TARA Transcript Abundances

## Data Availability

The datasets generated during and/or analyzed during the current study are available from the corresponding author on reasonable request.
