# Supplementary material for: Aerobic bacteria produce nitric oxide via denitrification and promote algal population collapse
Source: ISME J. 2023 May 12;17(8):1167–83. doi: 10.1038/s41396-023-01427-8 (PMC10356946; doi:10.1038/s41396-023-01427-8)
Supplement: Supplementary file 2 — Supplemental Material [file 41396_2023_1427_MOESM2_ESM.docx]

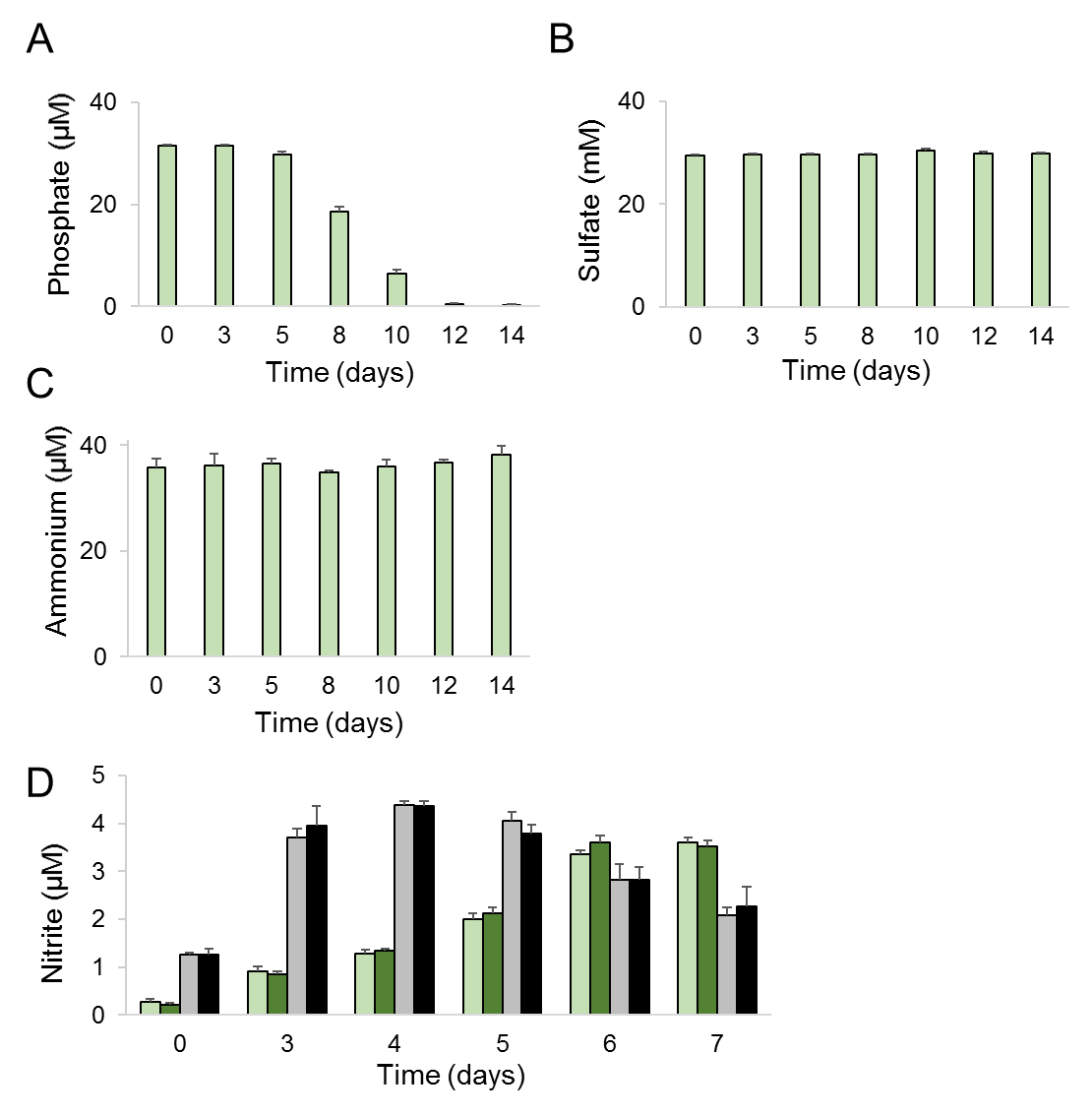
**fig. S1. Detection of inorganic molecules in filtrates of algal cultures.** (**A**) Phosphate (**B**) sulfate and (**C**) ammonium detected in filtrates of axenic algal cultures on the indicated days. (**D)** Nitrite levels measured in filtrates of axenic algal cultures (light green and gray bars) and co-cultures (dark green and black bars) inoculated with 10^3^ algal cells/ml (light and dark green bars) or 10^5^ algal cells/ml (gray and black bars). Statistical significance was calculated using a two-sample t-test to compare nitrite concentrations between samples inoculated with 10^3^ algal cells/ml versus samples inoculated with 10^5^ algal cells/ml. The difference between all samples was significant and resulted in p < 0.01. All results in the figure represent 3 biological replicates, error bars designate ± SD.


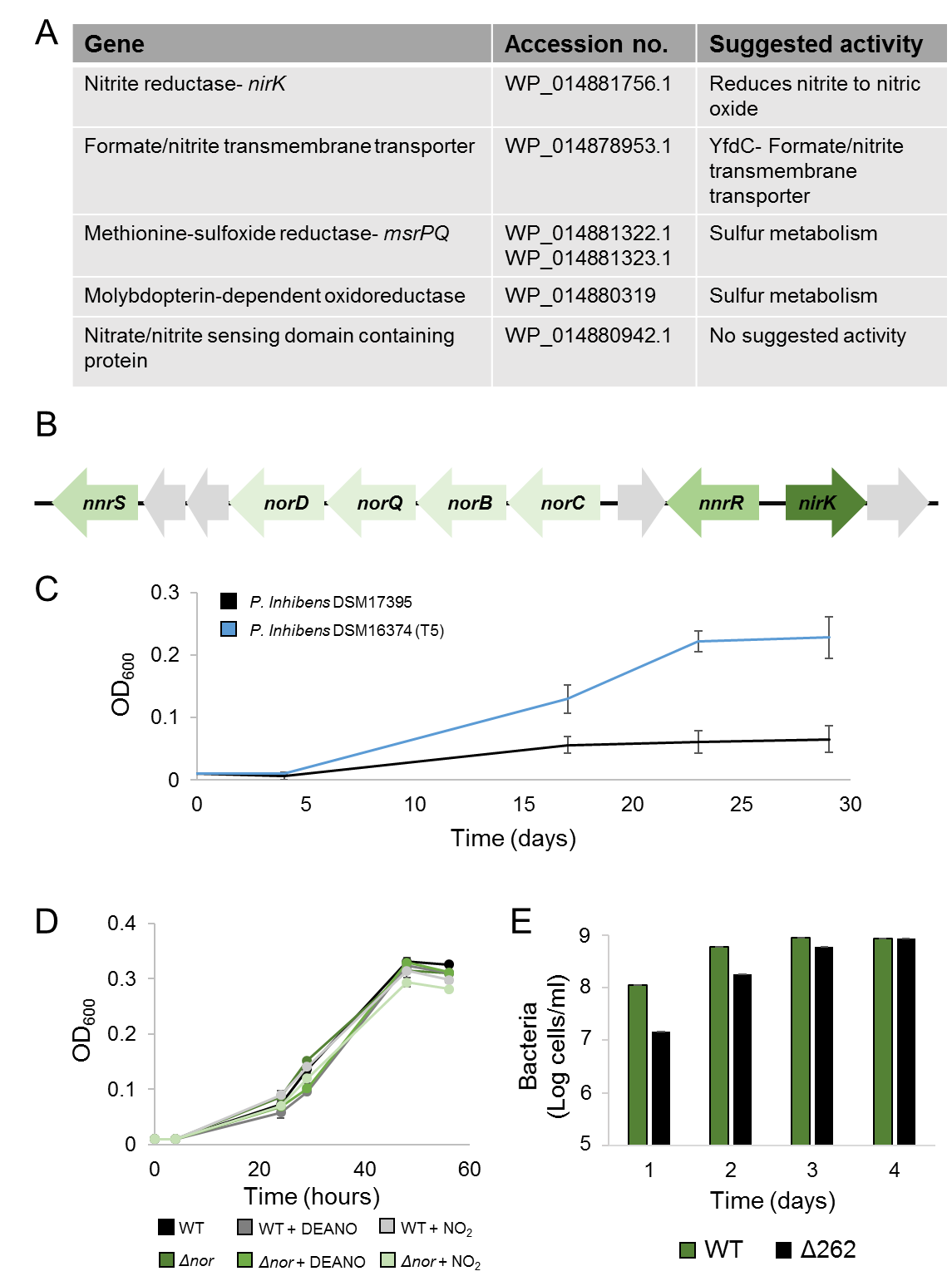


**fig. S2. Bacteria harbor denitrification genes but cannot grow under oxygen depleted conditions.** (**A**) Genes, accession numbers and suggested activities related to nitrite metabolism in the *P. inhibens* genome. Data compiled from the BioCyc database ^1^ and relevant literature ^2^. (**B**) Denitrification-related genes found in the native 262 kb plasmid of *P. inhibens*. Gray arrows- genes encoding hypothetical proteins, see table S2 for detailed description. (**C**) Growth of bacteria under anoxic conditions. Bacteria were inoculated into liquid medium with 10 mM NaNO_2_ and cultivated for 29 days under anoxic conditions. Shown are measurements of OD_600_ of *P. inhibens* DSM17395 (black line) and *P. inhibens* DSM16374 (T5) (blue line). Each data point consists of 3 biological replicates. Error bars designate ± SD. Statistical significance was calculated using a two-sample t-test to compare OD_600_ values between the two bacterial species at the different time points. The difference in OD_600_ values was significant for days 17, 23 and 29 and resulted in p < 0.05. (**D**) OD_600_ of WT and *Δnor* bacteria monitored during 56 hours with and without addition of 100 µM DEANO or 100 µM NO_2_. Each data point consists of 2 biological replicates, error bars designate ± SD. (**E**) Growth of WT (green bars) and Δ262 bacteria cured from the native 262 kb plasmid (black bars) in pure bacterial cultures. Each data point consists of 3 biological replicates, error bars designate ± SD. Statistical significance was calculated using a two-sample t-test to compare the cell count between the two bacterial species at the different time. The difference in cell count was significant for days 1 and 2 and resulted in p < 0.01.


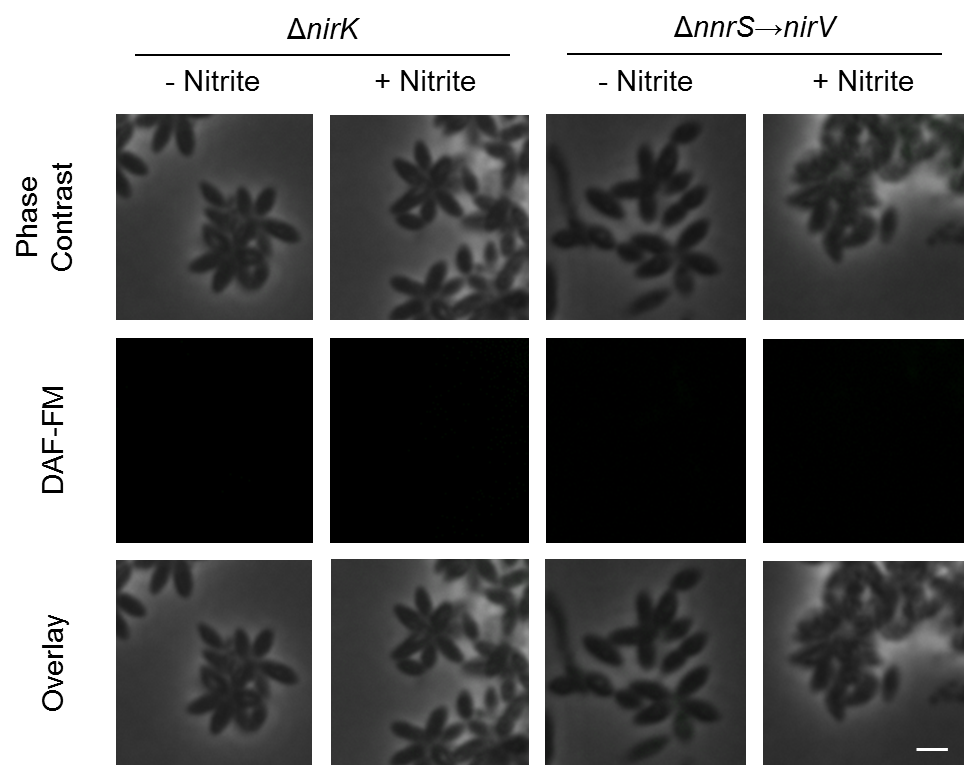


**Fig. S3. Intracellular NO in mutant bacteria.** Microscopy images of Δ*nirK* and Δ*nnrS→nirV* bacteria, stained with the fluorescent NO-indicator diacetate DAF-FM, and incubated with or without 100 µM nitrite for 2 h. Scale bar corresponds to 1 µm.

**
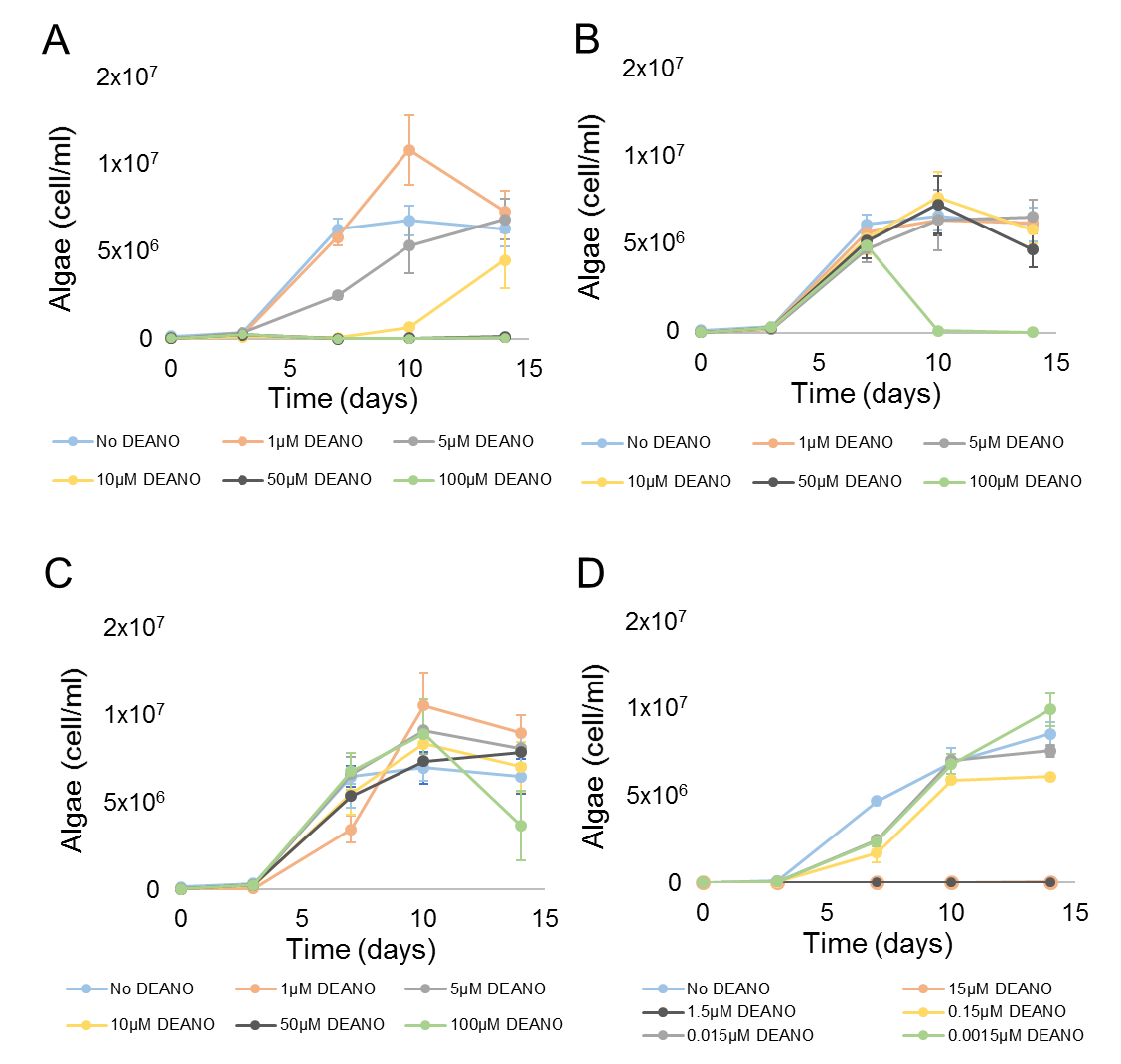
**

**fig. S4. The NO influence on algal growth.** Algae were subjected to various NO treatments and growth was monitored for 14 days. The NO donor DEANO was added at the indicated concentrations. The light blue line in each graph represents a control of no DEANO addition. DEANO was added as a single dose on (**A**) day 3 at the early exponential phase, (**B**) on day 7 at the late exponential phase, and (**C**) on day 10 at the stationary phase. (**D**) DEANO was added twice a day, every day, in the morning and afternoon. Each data point consists of 3 biological replicates. Error bars designate ±SD.

Statistical significance was calculated using a two-sample t-test to compare between algal cell counts in control versus DEANO treatments in all experiments. (**A**) On day 7, control versus 5, 10, 50 and 100 µM DEANO; on day 10, control versus 1, 10, 50 and 100 µM DEANO; and on day 14, control versus 50 and 100 µM DEANO had significant differences and resulted in p < 0.05. (**B**) Control versus 100 µM DEANO on day 10 and control versus 100 µM DEANO on day 14 had significant differences and resulted in p < 0.05. (**C**) Control versus 1, 5 and 100 µM DEANO on day 7 and control versus 1 and 100 µM DEANO on day 14 had significant differences and resulted in p < 0.05. (**D**) Control versus all DEANO concentrations on day 7 and control versus 0.15 µM DEANO on day 14 had significant differences and resulted in p < 0.05.


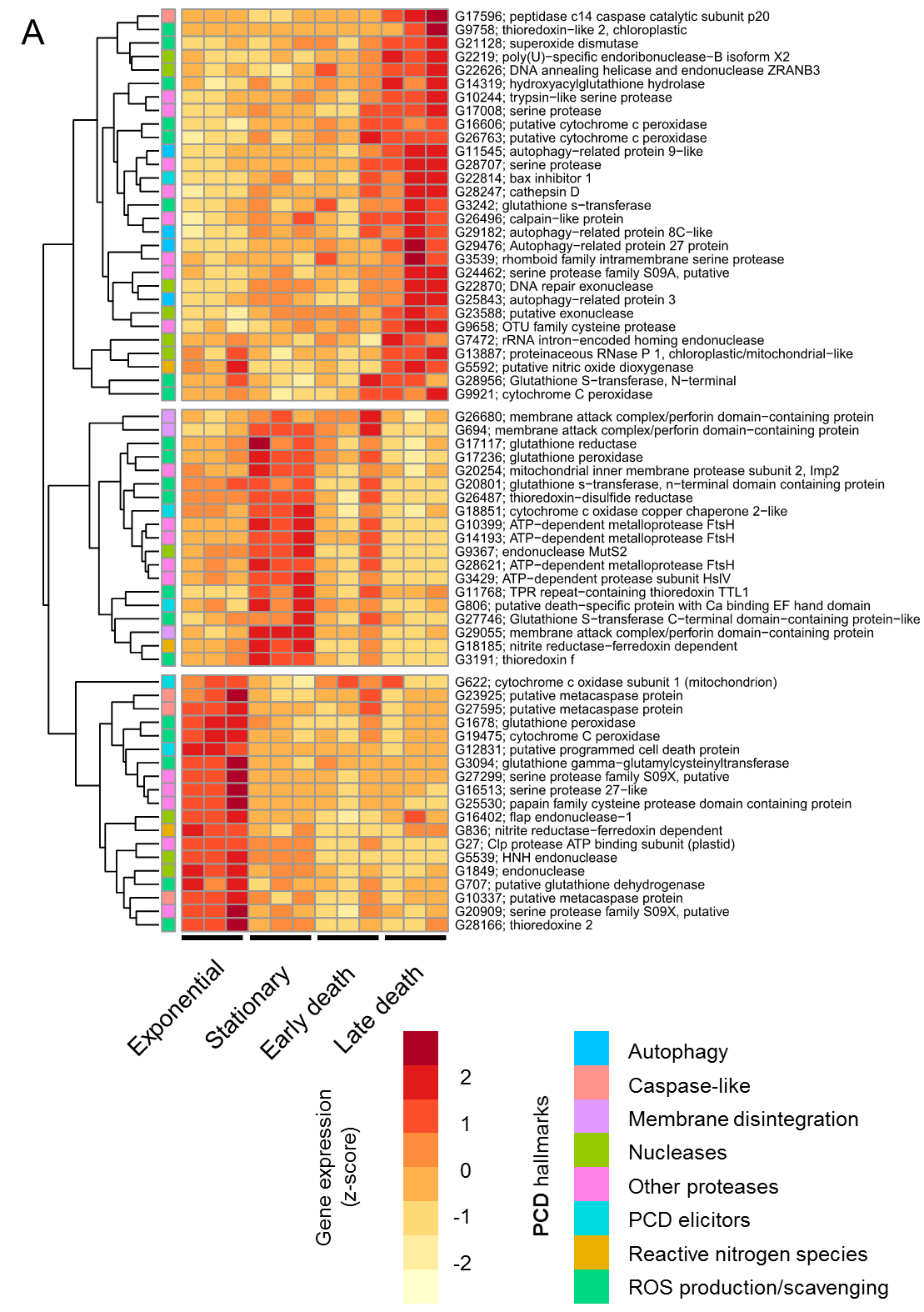


**
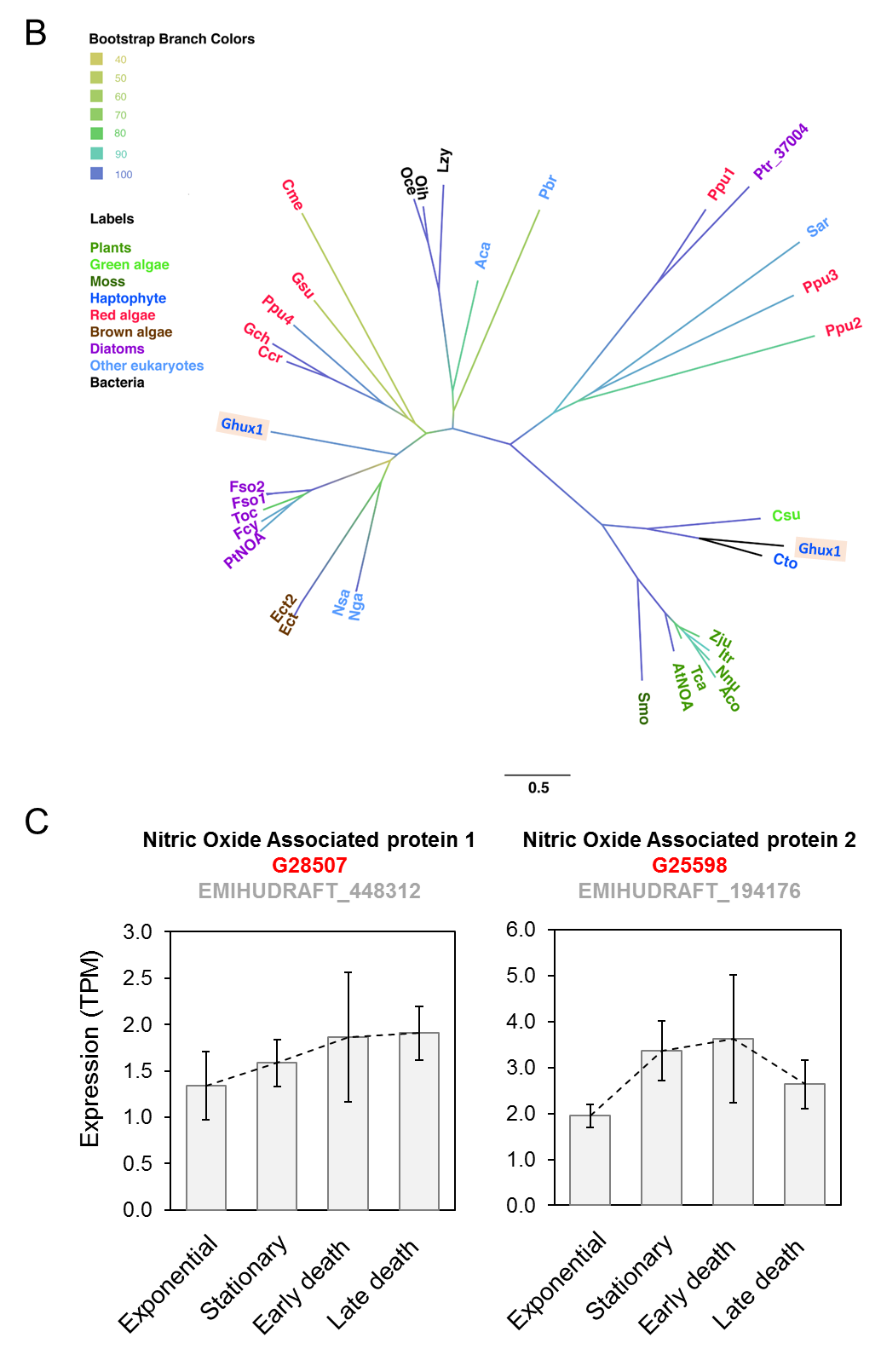
**

**fig. S5. Identification of PCD-like and oxidative stress related genes in algae.** (**A**) A heatmap showing the expression of putative *G. huxleyi* oxidative stress and PCD-related genes that were differentially expressed during the indicated four phases of co-culture (adjusted p value <0.1). Changes in gene expression are presented in red to yellow scale, with yellow indicating a decrease gene expression and red indicating an increase in gene expression. Genes were clustered according to expression pattern similarities. Gene IDs and product annotations are presented at the right side of the heatmap. Colored squares at the left side of the heatmap indicate suggested gene function. (**B**) Phylogenetic tree of Yqeh, the conserved domain in NO producing enzymes (see Materials and Methods). The branches are colored according to bootstrap, ranging from low (yellow) to high (blue). The species are colored according to phylogenetic range, see table S3. Plants- green, green algae- bright green, moss- dark green, haptophyte- blue, red algae- red, brown algae- brown, diatoms- purple, other eukaryotes- dark blue, bacteria- black. Tree scale: 1. (**C**) Gene expression levels of algal *noa* genes identified in (B) presented as transcripts per million (TPM), determined by total RNA sequencing on the indicated culturing phase. Gene annotations (bold), CCMP3266 gene IDs (red) and locus tag names of *G. huxleyi* CCMP1516 homologues genes present in the reference genome (gray). Expression data were taken from previously generated data ^3^. Each data point consists of 3 biological replicates, error bars designate ± SD.


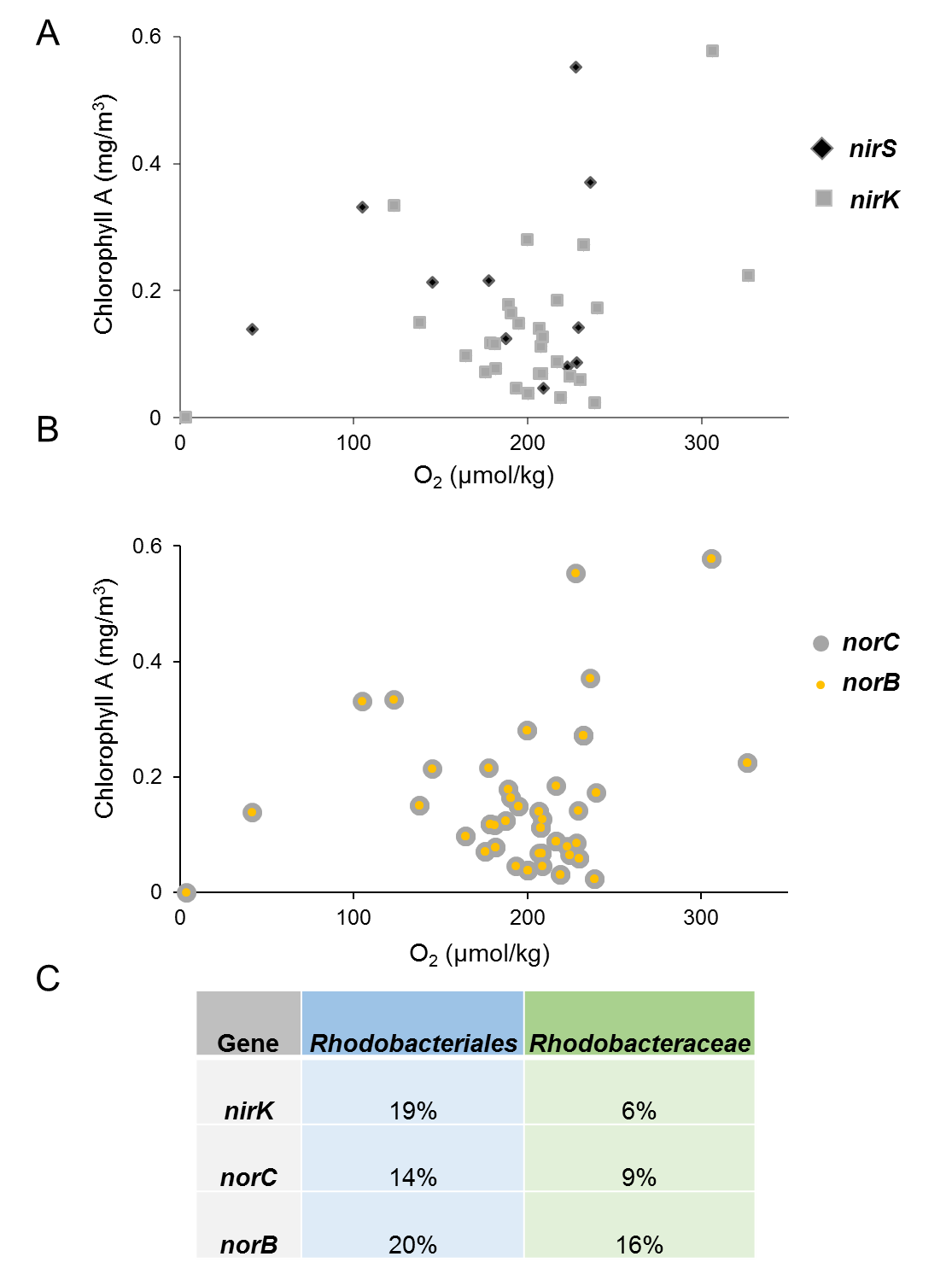


**
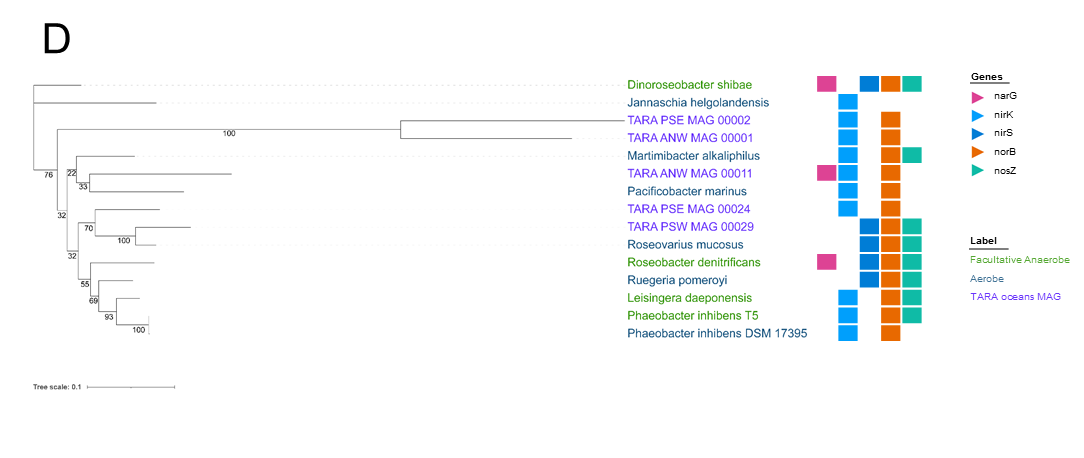
**

**fig. S6. Denitrification genes are found in deep chlorophyll maxima (DCM) regions in the ocean. (A-B)** DCM sampling points in which denitrification genes were detected are plotted against chlorophyll A and oxygen concentrations measured at the same location; (A) *nirS* and *nirK* (B) *norC and* *norB.* (**C**) Taxonomic distribution of the denitrification genes homologs detected in DCM sampling locations belonging to the *Rhodobacteriales* order and the *Rhodobacteracea* family. The location with the highest number of species detected was selected for presentation. (**D**) Phylogenetic tree of the *rpoB* gene from Roseobacter genomes of experimentally tested aerobes (table S1, species name in blue), anaerobes (table S3, species name in green) and Roseobacter MAGs (MAG identification name in purple). Bootstrap values are indicated at junctions. Identified denitrification genes in genomes are indicated in colorful squares to the right of each species. Pink- *narG* (nitrate reductase), light blue- *nirK* and dark blue- *nirS* (nitrite reductases), orange- *norB* (nitric oxide reductase), green- *nosZ* (nitrous oxide reductase). Tree scale: 0.1.


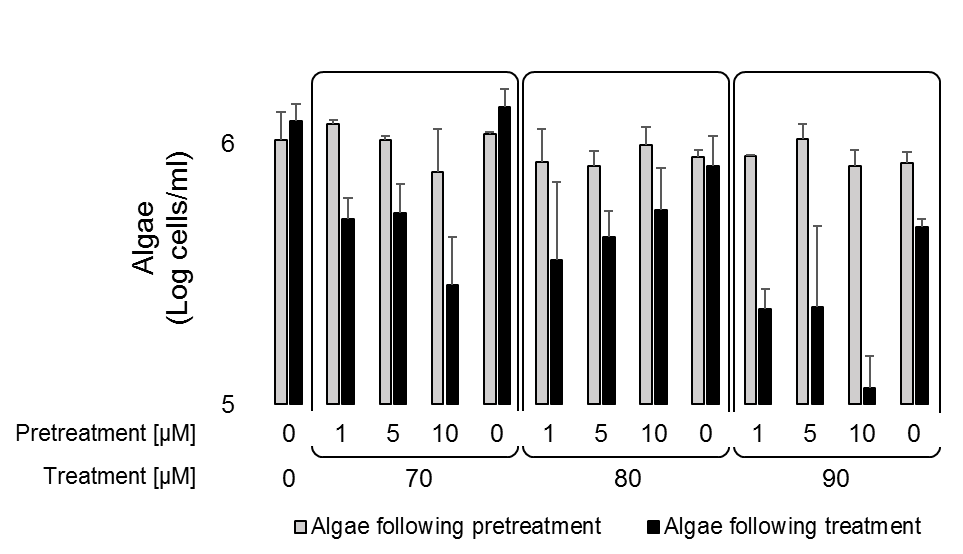


**fig. S7. Pre-treatment of algae with a sub-lethal NO dose results in increased susceptibility to NO.** Algae were pretreated with a single dose of 1, 5 or 10 µM DEANO on day 7 and algal cell numbers were monitored (grey bars). Two hours following pretreatment, algae were supplemented with a single dose of 70, 80 or 90 µM DEANO and algal cell numbers were monitored the following day on day 8 (black bars). As can be seen, pretreatment increased algal susceptibility to NO, and even sublethal concentrations (70 µM) induced algal death. Each data point consists of 3 biological replicates. Error bars designate ±SD. Statistical significance was calculated using a two-sample t-test to compare cell counts of algae before and after treatment. Pretreatment of 1 and 5 µM DEANO followed by 70 µM DEANO had significant differences and resulted in p > 0.05. Pretreatment of 1, 5 and 10 µM DEANO or no DEANO pretreatment followed by 90 µM DEANO had significant differences and resulted in p > 0.05.

| **Bacterial species** | **Location of isolation** | **Accession number** | **Reference** |
| --- | --- | --- | --- |
| *Jannaschia helgolandensis* DSM 14858 | Surface water sample, North Sea, Germany | *nirK* WP_092763076.1 | ^4^ |
| *Roseovarius*  *Mucosus* DSM 17069 | A dinoflagellate | *nirK* KGM86461.1 | ^5^ |
| *Martimibacter*  *alkaliphilus* HTCC2654 | Western Sargasso Sea | *nirK* TYP84150.1 | ^6^ |
| *Pacificbacter*  *marinus* CECT 7971 | Seawater sample, Yellow Sea, Korea | *nirK* WP_085849790.1 | ^7^ |
| *Rugeria pomeroyi* DSS-3 | Costal sea water | *nirS* [AAV97354.1](https://www.ncbi.nlm.nih.gov/protein/AAV97354.1) | ^8^ |
| *Phaeobacter*  *inhibens* DSM 17395 | Eukaryotic hosts and coastal biofilms^9^ | *nirK* WP_014881756.1 | Current study |

**table S1. Aerobic bacteria that carry denitrification genes.** Experimentally validated aerobic Roseobacters found in the literature, their location of isolation, identified denitrification gene and accession number and relevant reference.

|  | GeneID | Locus | ProteinID | GenBank Product |
| --- | --- | --- | --- | --- |
| 1 | PGA1_RS700 | NC_018291.1 | WP_014881747.1 | putative nnrS protein |
| 2 | PGA1_RS18705 | NC_018291.1 | WP_014881748.1 | hypothetical protein, similar to *nnrT* * |
| 3 | PGA1_RS18710 | NC_018291.1 | WP_014881749.1 | hypothetical protein, similar to *nnrT* * |
| 4 | PGA1_RS18715 | NC_018291.1 | WP_014881750.1 | putative protein NorD |
| 5 | PGA1_RS18720 | NC_018291.1 | WP_014881751.1 | putative protein NorQ |
| 6 | PGA1_RS18725 | NC_018291.1 | WP_014881752.1 | nitric oxide reductase subunit B |
| 7 | PGA1_RS18730 | NC_018291.1 | WP_014881753.1 | nitric oxide reductase subunit C |
| 8 | PGA1_RS18735 | NC_018291.1 | WP_014881754.1 | hypothetical protein ** |
| 9 | PGA1_RS18740 | NC_018291.1 | WP_036766773.1 | transcriptional regulator, crp family (nnrR) |
| 10 | PGA1_RS18745 | NC_018291.1 | WP_014881756.1 | copper-containing nitrite reductase NirK |
| 11 | PGA1_RS18750 | NC_018291.1 | WP_014881757.1 | hypothetical protein, similar to *nirV* *** |

**table S2: Denitrification-related genes on the 262 kb plasmid.** To identify denitrification-related genes on the native 262 kb plasmid of *P. inhibens* (accession: NC_018291.1) the plasmid was searched for genes that were previously annotated as involved in denitrification, and we examined the genomic locus in proximity to *nirK* (#10 in the table). Annotations were gathered from publicly available resources generated by Thole et al., 2012 ^10^, the NCBI Prokaryotic Genome Annotation Pipeline ^11^, KEGG ^12^ and UniProtKB ^13^.

* *nnrT*-like gene (61% and 68% identity to *nnrT* of *Salibaculum griseiflavum* and *Cohaesibacter sp. CAU 1516,* <https://www.uniprot.org/uniprotkb/A0A2V1P4H2/entry>, <https://www.uniprot.org/uniprotkb/A0A5R8YFT5/entry>.

** unknown

*** *nirV*-like gene (38% identity to *nirV* gene of *Ensifer adhaerens OV14*, <https://www.uniprot.org/uniprotkb/W8I8W7/entry>)


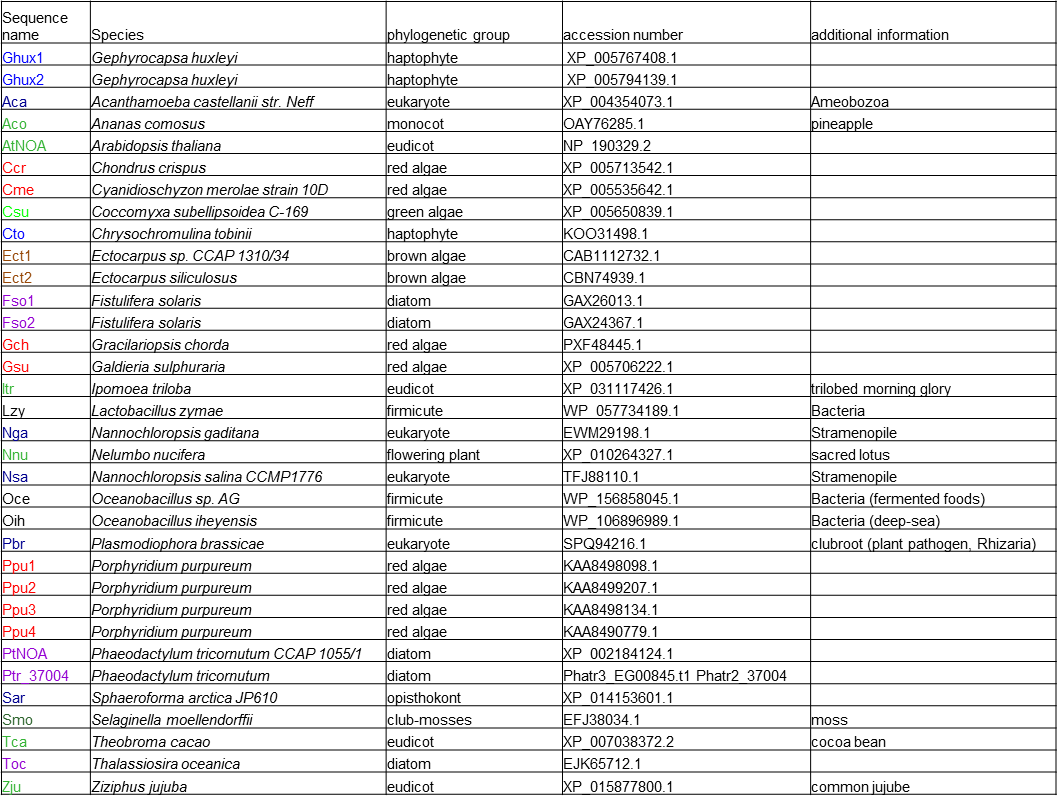


**table S3: Species that carry a Yqeh domain**. Species used to build the phylogenetic tree in fig. S5B. The name given to each species is color coded according to the phylogenetic range presented in fig. S5B. The phylogenetic group and gene accession number are also noted.

| **Bacterial species** | **Isolated from** | **Reference** |
| --- | --- | --- |
| *Dinoroseobacter shibae* FL12 | A dinoflagellate | ^14^ |
| *Roseobacter denitrificans* | Seaweed | ^15^ |
| *Leisingera daeponensis* | Tidal flat sediment | ^16^ |
| *Phaeobacter inhibens* T5 | German Wadden Sea | ^17^ |

**table S4. Facultative anaerobic Roseobacter bacteria.** Facultative Anaerobic Roseobacters used for building the phylogenetic tree in fig. S6D, their location of isolation and relevant reference.

**References**

1 Karp, P. D., Billington, R., Caspi, R., Fulcher, C. A., Latendresse, M., Kothari, A. *et al.* The BioCyc collection of microbial genomes and metabolic pathways. *Brief Bioinform.* 2019; **20**:1085-1093.

2 Wang, J., Krizowski, S., Fischer-Schrader, K., Niks, D., Tejero, J., Sparacino-Watkins, C. *et al.* Sulfite oxidase catalyzes single-electron transfer at molybdenum domain to reduce nitrite to nitric oxide. *Antioxid Redox Signal.* 2015; **23**:283-294.

3 Sperfeld, M., Yahalomi, D. & Segev, E. Resolving the microalgal gene landscape at the strain level: A novel hybrid transcriptome of *Emiliania huxleyi* CCMP3266. *Appl Environ Microbiol*. 2021; 88:**e**0141821.

4 Wagner-Döbler, I., Rheims, H., Felske, A., Pukall, R. & Tindall, B. J. *Jannaschia helgolandensis* gen. nov., sp. nov., a novel abundant member of the marine Roseobacter clade from the North Sea. *Int J Syst Evol Microbiol.* 2003; **53**:731-738.

5 Biebl, H., Allgaier, M., Lünsdorf, H., Pukall, R., Tindall, B. J. & Wagner-Döbler, I. *Roseovarius mucosus* sp. nov., a member of the Roseobacter clade with trace amounts of bacteriochlorophyll a. *Int J Syst Evol Microbiol.* 2005; **55**:2377-2383.

6 Lee, K., Choo, Y. J., Giovannoni, S. J. & Cho, J. C. *Maritimibacter alkaliphilus* gen. nov., sp. nov., a genome-sequenced marine bacterium of the Roseobacter clade in the order Rhodobacterales. *Int J Syst Evol Microbiol.* 2007; **57**:1653-1658.

7 Jung, Y. T., Lee, J. S., Oh, K. H., Oh, T. K. & Yoon, J. H. *Roseovarius marinus* sp. nov., isolated from seawater. *Int J Syst Evol Microbiol.* 2011; **61**:427-432.

8 Lee, K., Choo, Y. J., Giovannoni, S. J. & Cho, J. C. *Ruegeria pelagia* sp. nov., isolated from the Sargasso Sea, Atlantic Ocean. *Int J Syst Evol Microbiol.* 2007; **57**:1815-1818.

9 Gram, L., Rasmussen, B. B., Wemheuer, B., Bernbom, N., Ng, Y. Y., Porsby, C. H. *et al.* *Phaeobacter inhibens* from the Roseobacter clade has an environmental niche as a surface colonizer in harbors. *Syst Appl Microbiol.* 2015; **38**:483-493.

10 Thole, S., Kalhoefer, D., Voget, S., Berger, M., Engelhardt, T., Liesegang, H. *et al.* *Phaeobacter gallaeciensis* genomes from globally opposite locations reveal high similarity of adaptation to surface life. *ISME J.* 2012; **6**:2229-2244.

11 Tatusova, T., DiCuccio, M., Badretdin, A., Chetvernin, V., Nawrocki, E. P., Zaslavsky, L. *et al.* NCBI prokaryotic genome annotation pipeline. *Nucleic Acids Res.* 2016; **44**:6614-6624.

12 Kanehisa, M. & Goto, S. KEGG: kyoto encyclopedia of genes and genomes. *Nucleic Acids Res.* 2000; **28**:27-30.

13 Morgat, A., Lombardot, T., Coudert, E., Axelsen, K., Neto, T. B., Gehant, S. *et al.* Enzyme annotation in UniProtKB using Rhea. *Bioinformatics.* 2020; **36**:1896-1901.

14 Wagner-Dobler, I., Ballhausen, B., Berger, M., Brinkhoff, T., Brinkhoff, I., Bunk, B. *et al.* The complete genome sequence of the algal symbiont *Dinoroseobacter shibae*: a hitchhiker's guide to life in the sea. *ISME J.* 2010; **4**:61-77.

15 Arata, H., Serikawa, Y. & Takamiya, K. Trimethylamine N-oxide respiration by aerobic photosynthetic bacterium, *Erythrobacter sp.* OCh 114. *J Biochem.* 1988; **103**:1011-1015.

16 Yoon, J. H., Kang, S. J., Lee, S. Y. & Oh, T. K. *Phaeobacter daeponensis* sp. nov., isolated from a tidal flat of the Yellow Sea in Korea. *Int J Syst Evol Microbiol.* 2007; **57**:856-861.

17 Dogs, M., Voget, S., Teshima, H., Petersen, J., Davenport, K., Dalingault, H. Genome sequence of *Phaeobacter inhibens* type strain (T5^T^), a secondary metabolite producing representative of the marine Roseobacter clade, and emendation of the species description of *Phaeobacter inhibens*. *Stand Genomic Sci.* 2013; **9**:334-350.
